# When proxy-driven learning is no better than random: The consequences of representational incompleteness

**DOI:** 10.1371/journal.pone.0271268

**Published:** 2022-07-13

**Authors:** Justin Zobel, Felisa J. Vázquez-Abad, Pauline Lin

**Affiliations:** 1 School of Computing & Information Systems, the University of Melbourne, Parkville, Victoria, Australia; 2 Department of Computer Science, City University of New York, New York, New York, United States of America; UCLA, UNITED STATES

## Abstract

Machine learning is widely used for personalisation, that is, to tune systems with the aim of adapting their behaviour to the responses of humans. This tuning relies on quantified features that capture the human actions, and also on objective functions—that is, proxies – that are intended to represent desirable outcomes. However, a learning system’s representation of the world can be incomplete or insufficiently rich, for example if users’ decisions are based on properties of which the system is unaware. Moreover, the incompleteness of proxies can be argued to be an intrinsic property of computational systems, as they are based on literal representations of human actions rather than on the human actions themselves; this problem is distinct from the usual aspects of bias that are examined in machine learning literature. We use mathematical analysis and simulations of a reinforcement-learning case study to demonstrate that incompleteness of representation can, first, lead to learning that is no better than random; and second, means that the learning system can be inherently unaware that it is failing. This result has implications for the limits and applications of machine learning systems in human domains.

## 1 Introduction

A wide range of computational activities are performed by systems that are based on machine learning. Some of these activities are designed to adapt or respond to human actions using reinforcement learning, thus modifying their behaviour to improve the experience perceived by the humans with whom they are interacting. Examples include email spam filters, search engines, and recommender systems. Some systems of this kind respond in the same way to all users, producing results aggregated across all interactions, while others are *personalised*, that is, they adapt to the behaviour of individuals.

Such systems must (obviously) operate within the constraints of a computational system. That is, they can only make use of the input with which they are provided; they must reduce human actions and real-world entities to abstract, literal representations; and they must use proxies to represent the desired outcome, which at the human level might be ‘happiness’–or *satisfaction*, the term we use in this work.

A computational proxy must be a quantified metric that is designed to correspond with satisfaction. Thus, for example, in web search a simple proxy for satisfaction might be ‘the proportion of times that the user clicks on the first item in the results list’, reflecting the intuition that a user is happy if the first answer appears to contain the information being sought. Actual user responses might be more diverse; for example, the user might not click at all if the document summary on the results pages result contains the required information.

The proxy could then be refined, using mouse hovers for example, but there will always be a gap between the computational proxy and the richness of human behaviours. That is, a click or a hover do not demonstrate satisfaction; these actions correlate with satisfaction but are not equivalent. Likewise, categories of newspaper article—such as politics, sport, world, or culture—are convenient and helpful, but can easily not align with the much richer features that attract user interest. We argue that it is inevitable that a proxy for such applications will not be perfectly aligned with human goals.

It is this gap that we describe as *representational incompleteness*. This problem is distinct from the kinds of *bias* that are commonly examined in machine learning, which concern issues such as poorly chosen training data, incorrect labelling, missing data, or unbalanced categories [1]; and is also distinct from other problems that arise in the more specific context of reinforcement learning, such as the echo chamber effect. In systems whose behaviour is driven by interaction, we show that proxy mismatch or mismatch between features known to the system and those of interest to the user [2]—that is, representational incompleteness–can lead to poor outcomes even if the usual forms of bias are absent.

### 1.1 Contribution

In this paper, we explore representational incompleteness in the context of personalisation based on reinforcement learning, by examining behaviour where the representation is incomplete or the proxy does not fully capture human goals. To our knowledge this is the first exploration of the implications of this innate limitation of learning systems. Our work studies a core element of reinforcement learning, via specific scenarios in which misinterpretation of user feedback misleads the system. Due to the fact that human behaviour and real-world entities are rich and not amenable to complete description, some human behaviour will not be fully captured and some proxies will be inadequate, so that some level of misinterpretation is inevitable. We argue that a consequence of these factors is that the algorithm cannot discover that it is not performing well, and that this is an inherent challenge across a wide range of applications of machine learning, even for systems that are believed to work well.

We again note that these issues are distinct from challenges such as fairness, insufficiency, or errancy of the data, which also lead to failures of learning but through different mechanisms and with different effects. Moreover, while it may be common knowledge that computational proxies are often incomplete and, for applications of machine learning that are not based on interaction, that the existence of incompleteness may limit the effectiveness of the methods, in such case the learning is still better than random. In contrast, as we show, there may be no improvement compared to random in the environment that we study.

As a case study we use a movie recommendation system that is similar to systems that are regarded as successful in other literature. Recommenders are systems in which the set of features (the representation) over which the learning takes place is limited to literal features; movies are typically described by a category and perhaps additional metadata such as year, cast, or production crew, while human preferences may take into account a much broader range of values. In such systems, the user may be unaware that the system is algorithmically learning from their behaviour.

Note, however, that we do not seek to contribute to the literature on effective recommendation—the use of these systems in our work is purely as a tool for exploring a more general principle. Note also that we do not inspect every form of recommender or every possible adjustment that could be made to rectify specific errors; the issues we highlight may take different forms in different scenarios, but our point is that the issues can arise, not the specific forms that they take.

We use a rigorous mathematical argument, supported by simulations, to show that a plausible system can produce poor results in response to reasonable sequences of human actions, as an inherent consequence of the fact that there are human motivations of which the system is unaware. We examine two cases: first, where the user’s behaviour is reasonable but has not been anticipated in the system design; second, where the user’s goal is not reflected in the proxy due to the system having incomplete semantics. These examples are used and argued for in recommendation systems literature. However, as we show, the chosen proxy can lead to severe performance issues. Representations of human semantics are by their nature incomplete, and thus, we argue, instances of such situations are inevitable.

An intuitive assessment could be that an ML system with an insufficiently rich proxy might in the worst case tend to be random, and indeed we show this to be case. Under reasonable assumptions for proxies and completeness of data representation, and even in the absence of bias, the performance of the system can systematically be random, or in extreme cases even worse.

It could be argued that this observation is obvious, but a key insight here is that the problem is inherent when there is a mismatch between a system’s representations and the richness of reality. Addition of features cannot in general resolve the difficulties: if there is a human element of which the system is unaware, its proxies can tend to indicate that it is doing well when in fact the representational gap is creating gross error. This outcome can occur even if the human is not intentionally acting in an adversarial way. Clearly any specific system limitation, once known to the developers, can be addressed by altering the system; the issue we are highlighting is that it may be impossible to recognise the limitation automatically.

We have not sought to solve this problem, but to demonstrate that the pathology we have identified is undiscoverable (by automatic means) when it occurs. It is obvious that a method with, say, uninformative features can be poor. However, as we show, even methods that have been argued to be strong can fail in the presence of unanticipated behaviour. Our results have implications for any application of machine learning that is designed to adapt to human behaviour and place limits on the claims that can be made for system performance: a hidden assumption is that the representation is sufficiently complete and that the proxy is sufficient, and yet in practice they cannot be. Thus, in some situations, such systems must silently fail.

## 2 Motivation and background

Machine learning has a range of well-understood limitations. One that has had significant recent attention is bias and fairness. We discuss these two areas below, to set context but also to demonstrate that representational incompleteness is distinct from the forms of bias that have previously had attention. We then review work on recommender systems, which we examine as a case study, again with a focus on bias and fairness.

### 2.1 Fairness

Suresh and Guttag [3] characterise the issue of fairness as ‘unintended consequences’ of machine learning, a scope that includes our work. Their taxonomy (and their integration of previous taxonomies) rests on an abstract description of machine learning as an aggregation of data collection and preparation followed by model development, evaluation, postprocessing, and deployment. This careful structure provides an effective framework for evaluation of the form and effect of failures; however, their use of the framework is limited to biases, and does not encompass the challenges we consider of incomplete representation of data or misinterpretation of human behaviour. Our work could naturally be included in an extension of Suresh and Guttag’s framework, as could other limitations such as model adaptation or translation, as discussed by Danks and London [4], which we consider further below. Specifically, for example, while several works consider the impact of data labels that are inaccurate, or of unrepresentative sampling of the data population, to our knowledge there has not been previous examination of the impact of data labels that are inadequately rich.

An overview and formal description of fairness in decision making is given by Mitchell et al. [1]. This work sets out the range of factors that can influence or undermine fairness; we now discuss some of these but note that their work illustrates that bias and fairness are closely related.

One of the factors they consider as a confound to fairness is the process for collection of the data, which can be flawed if it is drawn from an unbalanced population; for example, if the desired outcome is understanding of the prevalence of a disease, but the data is drawn from the set of visits to a medical specialist, then prevalence will be overrepresented. Another factor is algorithmic bias, where for example more features might be used for women than for men because women can be pregnant, thus omitting, for men, potential predictive values. Yet another factor is a false assumption that outcomes are independent; for example, treatment resources may be finite, so one person’s hospitalisation can mean that another person is not treated. A further factor is the assumption that a simple or prelimary indicator–a proxy, in our terms—adequately represents a desired outcome; for example, discharge from hospital does not necessarily mean that the individual has fully recovered. These issues arise even if there is no incompleteness in the data representation. (These examples are ours; Mitchell et al. give similar examples drawn from other domains.) This work is discussed further below.

Joseph et al. [5], also discussed in more detail below, demonstrate grounds on which a specific class of algorithm can be provably fair, within the constraints of a system that can ‘know’ whether the desired outcome has in fact been achieved, an issue they do not examine, and which constitutes a more fundamental limitation in that it relates to the question of how completely a computational system can interact with human behaviour.

### 2.2 Bias

Danks and London [4] examine bias from a principled perspective, providing a taxonomy of causes of algorithmic bias. It can arise from choice of training data, incorrect use of attributes or annotations, algorithmic failure, inappropriate generalisation, or misinterpretation of outcomes by the user. In earlier work, Calders and Žliobaitė [6] reviewed how unbiased procedures can have biased outcomes if there is a mismatch between data, labelling, and assumptions being made in the system development process.

These taxonomies provide a valuable framework for examination of the appropriateness of ML for specific tasks, though from our perspective they are incomplete. As a reflection on these codifications of bias, it is apparent that there isn’t universal agreement on definitions of bias or when it is of concern.

Additional forms of (undesirable) bias in machine learning arise when the underlying data contains errors or omissions; is insufficient in volume; or is poorly sampled, that is, some classes are overrepresented and others absent or inadequate, as noted by Mitchell et al. [1] in the context of fairness. Some forms are particular to reinforcement learning, for example, when system behaviour adapts to the users who choose to continue with the system but does not recognise the shortcomings that led to other users to abandon it, or learn how to better interact with these users. These are fundamental challenges in machine learning and outside the scope of this paper.

Of more direct relevance to our work is that of Haug et al. [2], who consider the behaviour of learning-by-feedback when there is a mismatch between the features captured in the system and those of interest to the user. The focus is a specific application of learning in which a system is being explicitly trained by an individual to perform well at a defined task. They demonstrate that in such contexts, the reward function—proxy, in our terminology—may not align with the ‘true’ reward being sought, and propose ways in which a system can be designed to allow greater flexibility in training once a failing is discovered. The contrast with our work is that, in our analysis, we do not assume that the user is aware that learning is taking or place or has an opportunity to modify the learner’s behaviour, while their work assumes that there is a mechanism for observing that a failure has occurred. That is, the generality of the problem of failure and its discovery is not examined.

In a similar vein, Wang et al. [7], proceeding from the observation that failure can occur due to mismatch, consider how communication with the user can be designed to allow the failure to be efficiently rectified. The underlying challenge of whether failure is discoverable is not examined.

### 2.3 Recommender systems

The kind of tool based on machine learning that we examine, as a case study, is recommender systems. These are designed to learn users’ preferences in order to *serve* a list of items for the user to choose from. This mechanism is designed to satisfy each user. However, this innately human outcome is not directly observable by the system, so a proxy must be used instead.

As an illustration, suppose that the server is an online provider of video entertainment. In this illustration, the items being served belong to various *categories* of movies; only rarely will a human consume the same item more than once, and thus it is preferable to always serve new items. The set A represents a finite but large set of categories of movies. We assume that there are always new (unseen) movies in each category a∈A.

A comprehensive survey of recommender systems [8] uses four state-of-the-art algorithms that are statistical methods for static analysis (supervised learning), though reinforcement learning algorithms, which we study, are not considered. Jannach and Jugovac [9] examine how recommender systems contribute to the environment in which they operate, such as an online business. While this is not the same question as how well the system is working, they contrast proxies with measures such as how the recommender contributes as assessed by value to the business, and examine ways in which measures can fail due to bias or to uncertainty in how data was gathered.

Chaney et al. [10] examine a form of failure that is of relevance to our work, namely how the output of a recommender system (a collaborative filter, in contrast to the single-user modelling that we consider) can alter the user’s responses, thus leading to undesirable system behaviour. As in our investigations, recommendation is used as a case study of a broader principle. In Chaney et al., the principle is that the apparent success of a system can be due, not to delivering what a user desires, but instead, in effect, to changing the user. This can be regarded as creating a bias in the user population, but although there are similarities to our work this result does not relate to representational incompleteness.

The failure examined by Chaney et al. is that the system outputs converge to a homogeneous norm—an outcome that is arguably better than the behaviour we observe, that outputs can be random or worse—but still far from desirable. The cause and the effect of the failure is different but the approach they take has elements in common with ours, as they use simulations based on a rich user model of realistic actions. While our approach is by analysis, with the simulations primarily used to illustrate the correctness of our theoretical results, the work leads to similar observations—namely, that gaps in the assumed model in the system leads to unknown, undesirable system behaviours.

The case where a user’s interests, or preferences, change over time is studied by Jiang et al. [11], where a single user’s behavior is studied through a feedback-loop mathematical model. Specifically, they consider a multi-armed bandit (MAB) problem with multiple choices (arms) selected at each iteration. The user then acts and a ‘reward’ is provided to the computer in order to update its recommendation. They show that under some reasonable settings the model, a particular case of reinforcement learning, will tend to degenerate and create a ‘filter bubble’ effect.

In work by Joseph et al. [5] and Mitchell et al. [1], similar models are used to illustrate the effect on hiring policies of demographic categories. The focus of these papers is fairness, and they further use the concept of a context, which is an additional descriptor. In the hiring scenario, categories partition a population using, for example, ethnicity, while the descriptor contains information particular to the job qualification of the applicant. Mitchell et al. argue that categorisation of assumptions and choices can mitigate the problems, but there is ongoing conflation of technical and social contributions to the failings of ML, while also arguing that precise definitions of the challenges remain elusive. (Mitchell et al. make use of and cite a deep body of academic literature and public commentary on fairness in ML, both from a technical and social perspective. This material is for the most part beyond the scope of our work but is a notable resource on the topic.) Implicitly, they identify that assumptions and inherent technical constraints are critical to the question of whether ML can be effective.

In Joseph et al. [5], the MAB is designed to choose the best category, while ensuring that, with high probability, applicants that rank better with respect to descriptors will not be discriminated against. They also use a reinforcement learning (RL) algorithm that is a modification of UCB, with fairness explicitly defined in terms of the context. Fairness and optimization are then balanced using a trade-off cost that includes the context and the perceived reward. That is, the goals of the algorithm are completely specified, or in our terminology the proxy is assumed to be perfectly aligned with the human goals.

## 3 Case studies of failure to learn

We explore, using theoretical models, cases where reinforcement learning is expected to fail. As an illustration we focus on recommender systems, because they are an example of a general, ubiquitous technology that is well understood, but we do not propose refinements to recommender systems or contrast recommender technologies—this paper is not about improvements in recommendation. Our contribution is to demonstrate that, under realistic assumptions, *in the presence of unknown objectives or incomplete object representations it can be inevitable that first, learning will fail, and second, that the failure is invisible to the system*.

The taxonomy of bias by Danks and London [4] does not include the causes of failure that we examine: incompleteness of descriptions of the data and algorithmic misinterpretation of the user’s behaviour. That is, in contrast to the kind of bias-based failures that previous works have focused on, which arise because systems are vulnerable to learning and amplifying human discrimination, the failures we consider arise because computational systems are incomplete as representations of human activity.

We use the same model as Jiang et al. [11] to explore the phenomenon that even with a reasonable proxy, and in the absence of bias, some users may be persistently served items that decrease their satisfaction. This applies under the same design of the algorithm as in Jiang et al, including the ‘top-*ℓ*’ policy and the widely used Upper Confidence Bound (UCB) algorithm. We use simulations to illustrate and confirm the theoretical results, but it is the theory that is the core of the contribution.

We structure this study in three parts. In Section 3.1, we introduce a recommender system and a simple user model, and show that it behaves as expected. Our results follow from a class of MAB algorithms called *ϵ*-greedy, introduced by Sutton et al. [12] and provably convergent to the optimal solution, which we study because they are relatively easy to analyse mathematically. It is our view, however, that the principles behind our results are independent of the particular algorithm and therefore can arguably be extended to more complex models.

In Section 3.2 we extend this simple model to a case where the user does not know the labels for the items they are interested in, and exhibit behaviour that the designers of the system had not anticipated. Our results show that the true behaviour is drastically different from the system’s understanding of the behaviour, which is wildly overoptimistic.

In Section 3.3 we explore a case where the representation is incomplete in a simple way. Specifically, we assume that a user may be seeking a category that is not in A. We have discussed the need for a proxy to represent satisfaction, which is intended to be a measure of the quality of the output of a system. The representation used to describe the items is also a proxy. It is inherently incomplete and some human perspectives will inevitably be omitted. An example of representational incompleteness is a hidden or missing category. We show that the hidden category is undiscoverable, and leads to behaviour that is no better than random.

At first glance, our model for hidden or silent categories could be seen as similar to that of Joseph et al. [5], as they examine a characteristic of the bandit’s ‘arms’ that is not originally considered in the model for the categories. However, in their work the context is known in advance and is thus neither hidden nor silent. Incompleteness of the representation is fundamental to our observations, and to our knowledge has not previously been examined in this way as cause of failure in machine learning.

### 3.1 A simple recommender system

The case study we use throughout this paper is an elementary, but plausible, recommender system. We now present our model for such a system, which learns from a user’s preferences to make new suggestions. Here, and throughout the paper, the learning is based on the actions of a single individual rather than on a community of users and the system has no prior knowledge of the individual.

Recommendation items are drawn from a set. Each item in the set belongs to one of a fixed number of *categories*. The recommender system, or *server*, is an algorithm that is designed to choose the best categories for each user. The set of the labels of the categories is denoted by A={0,…,A-1} and contains A=∥A∥ categories. The recommendation *strategy* is defined as a policy $a_{t}=\left(a_{t,1},\ldots ,a_{t,\ell }\right),\;a_{t,k}\in A,\;\text{for}\;k=1\ldots ,\ell .$ where *t* is time (or iteration) and the user is being offered a list of *ℓ* items, where *ℓ* is constant. Each item *m* is represented by the category *a* from which it is drawn. The list of *ℓ* items served at time contains items (movies), one from each of the *ℓ* chosen categories. When greater specificity is required, that is, we need to know which items as well as the categories they are drawn from, we denote the actual list by ***m***_*t*_ = (*m*_*t*,1_, …, *m*_*t*,*ℓ*_); *m*_*t*,*k*_ ∈ *a*_*t*,*k*_ *fork* = 1…, *ℓ*.

Initially, we consider a model where the strategy aims to choose the ‘top-*ℓ*’ categories, ranked according to the user’s preference. Once ***a***_*t*_ is chosen, the items (for example, movies) are picked at random from each of the chosen categories. The user has an innate function, or behaviour, that represents their interest in each item (or category):

$$\begin{array}{c} \mu (a)=P(\text{user}\;\text{likes}\;\text{item}\;m\in a);\;a\in A. \end{array}$$

This function is not known, so the server must estimate it in order to attempt to serve a list that maximises user satisfaction.

Remark: In order to simplify notation, when only the categories rather than the actual items are relevant for the calculations we use the strategy ***a***_*t*_ in the analysis. It is assumed that the actual served list ***m***_*t*_ is characterized by the strategy ***a***_*t*_.

The cumulative reward *R*_*T*_ is defined by

$$\begin{array}{c} R_{T}=\sum_{t=1}^{T}\sum_{a\in a_{t}}\mu \left(a\right) \end{array}$$ (1)

The formulation of the problem is as follows: find the sequence {***a***_*t*_}, *t* = 1, 2, …, that maximizes expected long term satisfaction, that is:

$$\begin{array}{c} S=\underset{\left\{a_{t}\right\}}{\text{max}}(\underset{T\to \infty }{\text{lim}}\frac{R_{T}}{T})\overset{\text{def}}{=}\underset{\left\{a_{t}\right\}}{\text{max}}(\underset{T\to \infty }{\text{lim}}\frac{1}{T}\sum_{t=1}^{T}\sum_{a\in a_{t}}\mu \left(a\right)\;) \end{array}$$ (2)

The quantity *R*_*T*_ is the *expected cumulative reward* up to time *T*. The control problem in Eq (2) requires establishment of a policy where the list ***a***_*t*_ can only use information known to the server up to time *t*. Because *μ*(⋅) is not known, the server must learn it as the user provides feedback from the sequence of lists that have been presented to them. As is fundamental in machine learning, the server must use a proxy to learn the user’s preferences about the categories. Following Jiang et al. [11], we use clicks as the feedback, and denote as *c*_*t*_(*a*) the indicator function, meaning that the user clicks on an item in the served list that belongs to category *a*. This binary quantity is represented as *c_t_*(*a*) = **1**_{user clicks on item *m* ∈ *a*}_ for ***m*** ∈ *m*_*t*_, where **1**_{*q*}_ = 1 if *q* is true, and 0 otherwise (indicator function).

Remark Mathematically, {***a***_*t*_} must be a stochastic process adapted to the filtration of the user’s behaviour defined by consecutive clicks on items served, that is, ***a***_*t*_ may depend on the history of observations up to time *t*: {*c*_*k*_;*k* = 1, …, *t*}. Recall that the server does not know *μ* so it must estimate it in order to attempt to solve Eq (2). This problem is an example of a MAB problem, where multiple arms can be pulled at each iteration.

#### 3.1.1 Assumptions and optimal policies

In the recent literature on this subject, it is often assumed that the number of clicks is a good proxy for user’s satisfaction with each served item a∈A (see Jiang et al. [11] and references therein). Specifically, in the work of Jiang et al. [11] the server postulates a simple *click model* for the user:

$$\begin{array}{c} P\left(c_{t}\left(a\right)=1\right)=E\left[c_{t}\left(a\right)\right]=\mu \left(a\right),\;\forall a\in a_{t}. \end{array}$$ (3)

In this model, the user’s liking of a category is described by the number of clicks on that category. That is, the sample average of clicks is a good estimator of the unknown satisfaction measure *μ*. Let

$$\begin{array}{c} T_{t}\left(a\right)=\sum_{n=1}^{t-1}1_{\left\{a\in a_{n}\right\}} \end{array}$$ (4)

count the number of times item *a* has been served before time *t*.

**Lemma 3.1.** *Under the assumption described in* Eq (3), *for all* a∈A *such that T*_*t*_(*a*) → ∞ *with probability 1 (w.p.1) the estimator*

$$\begin{array}{c} \theta_{t}\left(a\right)=\frac{1}{T_{t}\left(a\right)}\sum_{n=1}^{t-1}c_{n}\left(a\right)1_{\left\{a\in a_{n}\right\}} \end{array}$$ (5)

*satisfies* lim_*t*→∞_ *θ*_*t*_(*a*) = *μ*(*a*) *w.p.1*.

The proof is straightforward using the strong law of large numbers, given that the consecutive observations have the same distribution and are Bernoulli random variables, observing also that Eq (5) is the usual sample average. The use of the indicator function ensures correctness as *t* tends to infinity.

An optimal learning policy must use some combination of *exploration* (serving of items *a* that are not in the top-*ℓ* in *θ*_*t*_) and *exploitation* (serving the top-ℓ items according to the estimator *θ*_*t*_ of the user’s preferences). The server strategy ***a***_*t*_ contains either (i) the top-*ℓ* categories ranked according to the estimators *θ*_*t*_ in Eq (5), if iteration *t* is an exploitation, or (ii) a random choice of categories in A if iteration *t* is an exploration. Let *p*_*t*_ > 0 denote the probability that the action at time *t* is an exploration. From Lemma 3.1 it follows that, if *p*_*t*_ → 0, then the observed satisfaction will converge to the optimal satisfaction rate in Eq (2). Specifically, the actual cumulative reward (or true user’s satisfaction) up to time *T* and the actual satisfaction rate are:

$$\begin{array}{c} {\hat{R}}_{T}\overset{\text{def}}{=}\sum_{t=1}^{T}\sum_{a\in a_{t}}c_{t}\left(a\right);\;{\hat{S}}_{T}=\frac{{\hat{R}}_{T}}{T}. \end{array}$$ (6)

Lemma 3.1 then implies that ${\text{lim}}_{T\to \infty }{\hat{S}}_{T}=S$.

In the literature on MAB problems, strategies that balance exploitation and exploration aim not only at strategies that converge to the optimal reward (user satisfaction in our case), but seek to do so with the fastest convergence rate. Auer et al. [13] provide a summary of the main results that are now used.

As far back as 1985, Lai and Robbins [14] show that, in order to maximize the convergence rate—equivalent to minimizing the ‘regret’, defined in terms of the difference between the optimal satisfaction and the true satisfaction, that is, $\left(S-{\hat{S}}_{T}\right)$—the number of times a sub-optimal arm is chosen must increase only logarithmically in *t*. Intuitively this means that a fast exploration rate will force the algorithm to serve non-optimal arms too often, and a slow exploration rate will lead to slow learning. In the first (1998) edition of their book, Sutton and Barto introduced the *ϵ*-greedy policy, where exploration is performed with constant probability *ϵ*; this work has been improved in what is today the more standard reference [12]. Auer et al. [13] study various implementations that are provably optimal, because they satisfy the condition for optimal regret given by Lai and Robbins.

We use the most common two methods in this paper, for illustration. The first is a modification of the *ϵ*-greedy policy, called by Auer et al. [13] the *ϵ*_*n*_-greedy algorithm, where the exploration probability *p*_*t*_ decreases as O(1/t), which ensures the logarithmic increase condition necessary for optimality. The second is the Upper Confidence Bound (UCB) family of algorithms, also studied in detail in Auer et al. [13], where it is shown that they satisfy the optimality condition. UCB algorithms, as well as Thompson schemes, are more complex than *ϵ*_*n*_-greedy. They are based on statistical estimation of upper bounds that include both estimation of the true values *μ*(*a*) as well as an estimation of the confidence interval that uses the Chernoff-Hoeffding bound. When implementing these, exploration is a consequence of the uncertainty in the estimation of the values *μ*(*a*).

In order to focus on the main results of this paper, we use the simplest strategy that ensures convergence to the optimal value: other strategies may converge faster, but our observations can be extended to more complex algorithms. To illustrate the validity of our results for other schemes we include simulations using a UCB scheme. We remark here that the literature and results quoted above on MAB usually assume that only one arm is pulled at each iteration. In our model, we present a list of *ℓ* items, so it is as pulling *ℓ* arms simultaneously. The corresponding policies are either the ‘top-*ℓ*’ both for the *ϵ*_*n*_-greedy as for the UCB algorithms, or a choice of list that draws the categories with likelihoods proportional to the estimated values of {μ(a);a∈A}.

#### 3.1.2 Simulation

We now present simulation results for this first model, Model 1, assuming that the user follows the behavior in Eq (3). The simulation is used to illustrate how the estimated reward can converge to the optimal reward as the number of iterations grow. Shown in abstract as Algorithm 1, it uses a modification of the *ϵ*-greedy algorithm in Sutton and Barto [12], where exploration is done with decreasing probability *p*_*t*_. Following Auer et al. [13] the exploration probability is decreased as follows. Let *c* > 0, 0 < *d* < 1, *K* > 1, and define

$$\begin{array}{c} p_{t}=\text{min}(1,\frac{cK}{d^{2}\;t});\;t=1,2,\ldots . \end{array}$$ (7)

The algorithm works as follows. At iteration *t*, with probability *p*_*t*_ the strategy list is a random choice of *ℓ* different elements in A (exploration), or otherwise the top *ℓ* elements of *θ*_*t*_ are served. We simulate the behavior of the user assuming Eq (3), so that for each element of category ***a*** ∈ *a*_*t*_ presented in the list the click value is a Bernoulli random variable with parameter *μ*(*a*). Next, the estimates are updated using Eq (5). The algorithm includes the computation of the estimate ${\hat{R}}_{T}$ (see Eq (6)) of the true cumulative reward *R*_*T*_ defined in Eq (1).

**Algorithm 1** Simulation algorithm for Model 1.

Initialize (read) A,μ,ℓ.

$p_{1}=1,\theta_{1}\left(a\right)=0,a\in A$

**for** t ≔ 1 to T **do**

 *u* = Rand()

 **if** *u* ≤ *p*_*t*_ **then**

  Explore: pick a random list ***a***_*t*_.

 **else**

  Exploit: pick the top *ℓ* elements of *θ*_*t*_.

 User Feedback: for all ***a*** ∈ *a*_*t*_ generate *c*_*t*_(*a*)∼ Bernoulli(*μ*(*a*))

 Server update: *θ*_*t*+1_(*a*) using (5).

 Calculate true reward: ${\hat{R}}_{t+1}={\hat{R}}_{t}+\sum_{a\in a_{t}}\mu \left(a\right)$

 Update exploration probability *p*_*t*+1_ using (7)

**Fig 1:**
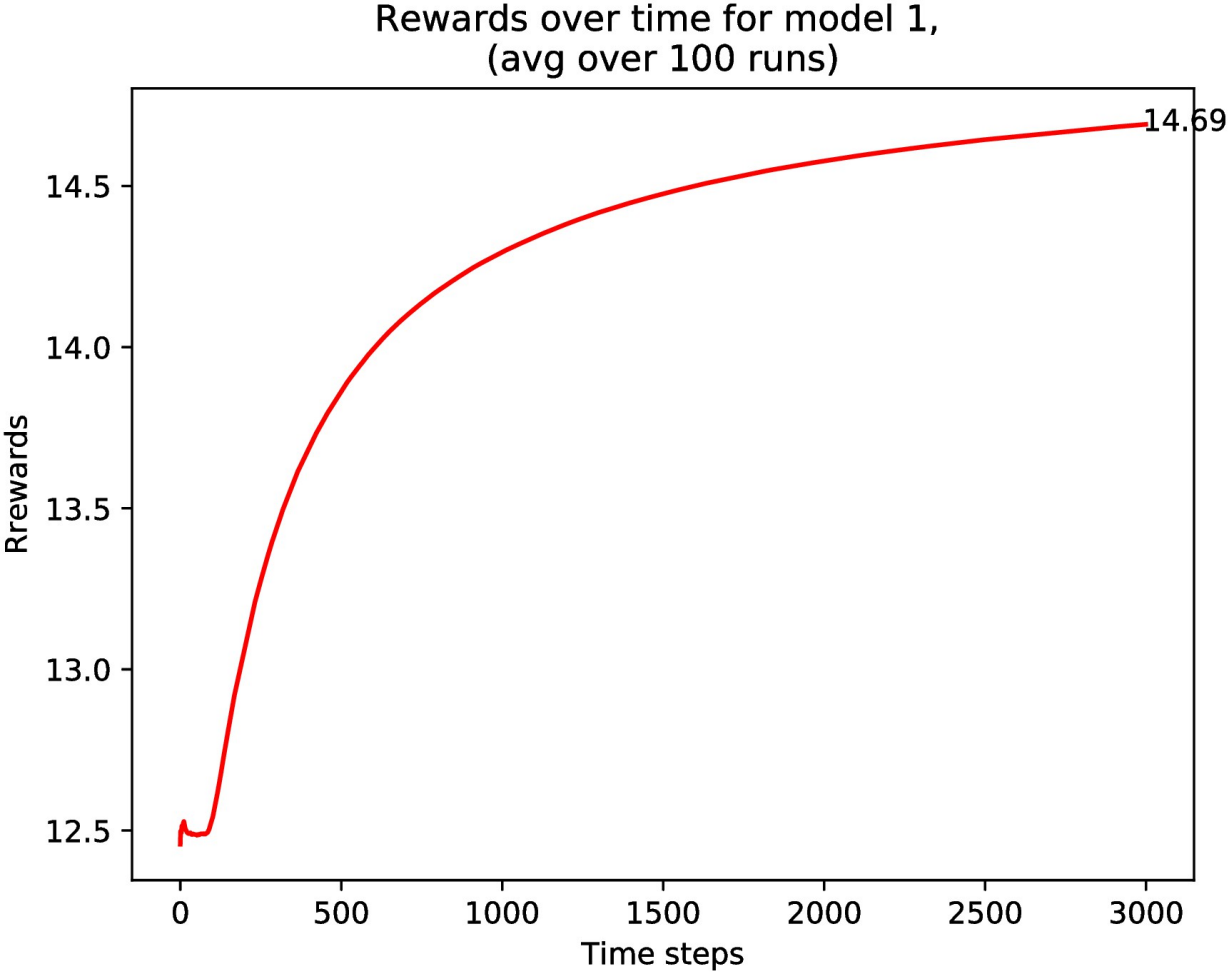
Simulation results for average reward rate function $({\hat{R}}_{t}/t),t=1,\ldots ,T$, where *A* = 100, *ℓ* = 20, *μ* = [0.75, …(×50), 0.5, …(×50)], and *T* = 1000, and $({\hat{R}}_{T}/T)=14.95$. The exploration rate is $p_{t}=\text{min}(1,\frac{cK}{d^{2}\;t})$ based on Eq (7) using *c* = 1, *K* = 5, and *d* = 0.25.

The outcome of the simulation is shown in Fig 1, in which we used *A* = 100 for the number of categories and and *ℓ* = 20 is the size of the served list. The true preferences vector is *μ*(*a*) = 0.75 for *a* = 0, …, 49 and *μ*(*a*) = 0.5 for *a* = 50, …, 99. Explaining these parameters, in the model the best reward is achieved by presenting *ℓ* = 20 items in any of the first 50 categories, and the corresponding satisfaction value is 20 × 0.75 = 15. Fig 1 shows how the learning approaches this limit. Note that it takes around 1000 steps to get satisfactorily close (within 0.05% error). This is significant because, in many applications, users may not be willing to wait that many steps before receiving good recommendations.

**Fig 2:**
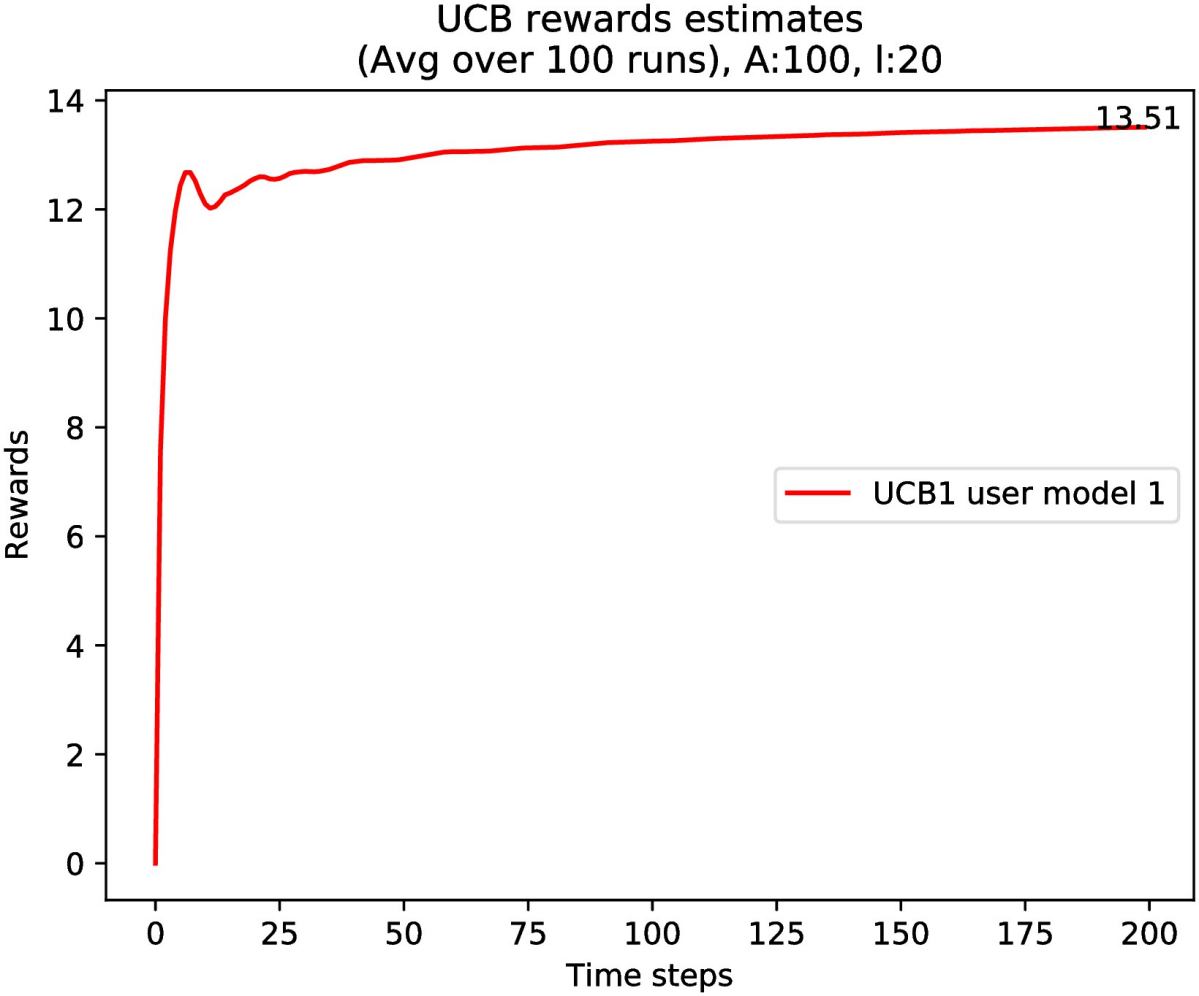
Simulation results for average reward rate function using the UCB algorithm $({\hat{R}}_{t}/t),t=1,\ldots ,T$, where *A* = 100, *ℓ* = 20, *μ* = [0.75, …(×50), 0.5, …(×50)], and *T* = 1000, and $({\hat{R}}_{T}/T)=14.95$.

For completeness, we present in Fig 2 the results of the learning recommender when using the UCB algorithm, which as discussed above is common in the literature of MABs. For our purposes, the UCB algorithm is adapted to the case when multiple arms are chosen at each iteration, because the recommended list contains *ℓ* items. As expected, UCB seems to converge faster than the *ϵ*_*n*_-greedy scheme illustrated in Fig 1, and it also has an asymptote of 15.

### 3.2 Realistic model for dissatisfied user

Consider now the following user model for clicking on items served. When presented with a list, in this model the user clicks on the items for inspection in the order in which they appear in the list. When an item is clicked, the user either watches the video or clicks on the next item, until finding one that they like. To illustrate the phenomenon of persistent error in recommender systems, consider now an unusual case: a user who happens to like only one category of movies $a^{*}\in A$, but does not know the label of the category. For example, it may be a rare combination of foreign-language dark romantic mini series. For this user, *μ*(*a*)≈0 for all categories *a* ≠ a*, whereas *μ*(a*) = 1.

In this example, both the system model and user model are simple, allowing us to analyse them mathematically. However, they are not unrealistic, and with large numbers of users of recommender systems some of the users will not be typical of the rest. The combination creates an interesting test case because of the likelihood that some behaviour will be unanticipated in the design of any realistic system. Thus it is a case where a proxy described in the literature does not capture a potential realistic human behaviour. As we show both mathematically and in simulation, this can lead to severe performance issues that cannot be detected by the system.

Here, then, the actual pattern of clicks is as follows. When served a list of items of categories ***a***_*t*_ = (*a*_*t*,1_, …, *a*_*t*,*ℓ*_) this user will click all items in order until either reaching an item in a* that is liked or until the list is exhausted. Formally, for ***a***_*t*,*k*_ ∈ *a*_*t*_,

$$\begin{array}{c} c_{t}\left(a_{t,k}\right)=\{\begin{array}{cc} 1 & \;\text{if}\;a^{*}\neq a_{t}\\ 1 & \;\text{if}\;\left(a_{t,j}=a^{*}\right)\;\&\;\left(k\leq j\right)\\ 0 & \;\text{otherwise}\; \end{array} \end{array}$$ (8)

Clearly $E[c_{t}\left(a\right)]\neq \mu \left(a\right)$, but the server continues behaving under the assumption of the model in Eq (3). The following analysis shows how this can lead to persistent recommender errors.

#### 3.2.1 Analysis and simulation results

Let *ν* = min(*t*:a* ∈ ***a***_*t*_) be the first time that the category a* is chosen by the server. Note that up to *ν* the user has clicked on all the items previously presented, so *θ*_*ν*_(*a*) = 1 for all served categories ***a*** ∈ {*a*_*k*_;*k* < *ν*} and *θ*_*ν*_(a*) = 0. When presented with the list from the strategy ***a***_*ν*_ the user clicks until finding an item in a*, so all items that appear in this list after that will decrease their estimate *θ*_*ν*_, and *θ*_*ν*+1_(a*) = 1. This stopping time *ν* is important. In particular, the true cumulative reward (satisfaction) for the user up to time *ν* is nearly zero: $\sum_{t<\nu }\sum_{a\in a_{t}}\mu \left(a\right)\approx 0$, whereas the server has used a model where the estimated cumulative reward is given by

$$\begin{array}{c} E[\sum_{t<\nu }\sum_{a\in a_{t}}c_{t}\left(a\right)]=\ell \times \left(E\left[\nu \right]-1\right), \end{array}$$

corresponding to a reward rate of approximately *ℓ* (the largest possible).

**Proposition 3.1.** *Let* A=|A| *be the size of the category set. Assume that the server chooses to explore at iteration t with probability p*_*t*_, *in which case* ***a***_*t*_ *consists of ℓ items chosen at random. Otherwise, the server presents the top-ℓ items according to its current estimate θ*_*t*_(⋅). *Then*

$$\begin{array}{c} E\left[\nu \right]=\sum_{k=1}^{\infty }\prod_{j=1}^{k-1}(1-p_{j}\frac{\ell }{A}). \end{array}$$ (9)

*Proof*. The proof is in an Appendix.

The preceding material allows us to make the following observations. First, for this scenario, the server estimates the asymptotic user’s satisfaction at 100%. This follows from the fact that the server believes model Eq (3), as set out in the simplistic model, while the true behavior is governed by Eq (8).

Second, if exploration is not done, then *p*_*t*_ = 0 and Proposition 3.1 is not applicable. In this case, unless a* ∈ ***a***_1_ is chosen for the first list, then necessarily the first *ℓ* categories chosen will have *θ*_1_(*a*) = 1 and will be persistently served for all time *t* ≥ 1. That is, only with small probability *ℓ*/*A* the user will be happy, but otherwise will be forever dissatisfied.

Third, if constant exploration is used (as in Sutton and Barto [12]) with *p*_*t*_ ≡ *ϵ* then *ν* is a geometric random variable with parameter (*ϵℓ*/*A*), so E[ν]=A/(∈ℓ). This number can be very large when *ℓ* ≪ *A*, leading to the server’s prediction error also being very large.

Fourth, even after time *ν*, when the server will estimate *θ*_*t*_(a*) = 1 for all *t* ≥ *ν*, there is a probability that the recommender system will continue to serve lists that do not contain items of category a* because many other items may also have an estimate *θ*_*k*_(*a*) = 1. If the lists are presented with the top *ℓ* items ordered at random, due to random exploration, the correct item a* will eventually *surface*, meaning that there is an a.s. finite time *k* such that $\forall a\in A,\;\theta_{k}\left(a\right)>0$ and *θ*_*k*_(a*)≥sup_*a*_(*θ*_*k*_(*a*)). From that point onwards a* will remain in the top *ℓ* of *θ*. Only in this scenario will the asymptotic true satisfaction be good.

Observe that, in all cases, the transition time until the recommender system begins to serve the preferred item a* with high frequency may be greater than is realistically reasonable.

**Proposition 3.2.** *Assume that when serving the top-ℓ list to the user, the server chooses the top ℓ categories according to θ*_*t*_, and under ties, it presents the corresponding items in the order of their category labels. Then if a* > ℓ the server will eventually stop serving category a* *during the exploitation iterations*.

*Proof*. The proof is in an Appendix.

As a corollary of the above results, the persistent errors yield asymptotically wrong recommendations under the assumptions of Proposition 3.2. In particular, ${\text{lim}}_{T\to \infty }\frac{1}{T}\sum_{t=1}^{T}\sum_{a\in a_{t}}\mu \left(a\right)\;=0$ with probability 1. To see this, write the expected reward up to time *T* (sufficiently large) as

$$\begin{array}{cc} \frac{R_{T}}{T} & =\frac{1}{T}\sum_{t=1}^{\tau }\mu \left(a\right)\;+(\frac{T-\tau +1}{T})(\frac{1}{T-\tau +1})\sum_{t=\tau +1}^{T}\mu \left(a\right) \end{array}$$

The first term will tend to zero w.p.1 as *T* → ∞. In the second term, the factor (*T* − *τ* + 1)/*T* → 1, and the other factor is an average reward. Thus, for large *T*, using exploration probability *p*_*t*_ = *ρ*^*t*^, so that the likelihood of exploration falls to 0 over time,

$$\begin{array}{cc} \frac{R_{T}}{T} & \leq (\frac{1}{T-\tau +1})\sum_{t=\tau +1}^{T}P\left(a^{*}\in a_{t}\right)+c\cdot T^{-1}\\ & =(\frac{1}{T-\tau +1})\frac{\ell }{M}\;\sum_{t=\tau +1}^{T}\rho^{t}+c\cdot T^{-1}\leq (\frac{\ell }{M(T-\tau +1)})\frac{1-\rho^{T}}{1-\rho }+c\cdot T^{-1}\to 0, \end{array}$$

where *c* is a constant. In the case of fixed exploration probability (*p*_*t*_ = *ϵ*), the corresponding limit average reward is *ϵℓ*/*A*, which is very small. In any case, the server’s estimate of the average reward will be 100% satisfaction. Therefore, even if *θ*_*t*_(a*) = 1 correctly estimates *μ*(a*), the error due to the wrong proxy will be persistent and will lead to near-complete dissatisfaction.

Remark: In this case, the server believes that the user’s actions satisfy Eq (3), so the server’s estimate for the believed reward is ${\tilde{R}}_{T}=\sum_{t=1}^{T}\sum_{a\in a_{t}}c_{t}\left(a\right)$, In fact, however, $E\left[{\tilde{R}}_{T}\right]\neq E\left[R_{T}\right]$.

To compare the behavior of the recommender system for a dissatisfied user with the behavior that the server is assuming, we perform the simulation shown in Algorithm 2. The only difference in the simulations is the behavior of the user, that is, the calculation of the click binary variables *c*_*t*_(*a*). Naturally the server does not know what model a user follows.

**Algorithm 2** Simulation algorithm for model 2

Initialize (read) A,ℓ,ρ<1, a*.

$p_{1}=1,\theta_{1}\left(a\right)=0,a\in A$ *μ*(a*) = 1; *μ*(*a*) = 0, *a* ≠ a*.

**for** t ≔ 1 to T **do**

 *u* = Rand()

 **if** *u* ≤ *p*_*t*_ **then**

  Explore: pick a random list ***a***_*t*_.

 **else**

  Exploit: pick the top *ℓ* elements of *θ*_*t*_ (randomize if more than *ℓ* are top).

 User Feedback:

 *j* = 0

 **while** (*a*_*t*,*j*+1_ ≠ a*) or (*j* < *ℓ*) **do**

  *j* ≔ *j* + 1

  *c*_*t*_(*a*_*t*,*j*_) = 1

 Server update: *θ*_*t*+1_(*a*) using (5).

 Calculate true reward: ${\hat{R}}_{t+1}={\hat{R}}_{t}+\sum_{a\in a_{t}}\mu \left(a\right)$

 Calculate the server’s belief about the reward: ${\tilde{R}}_{t+1}={\tilde{R}}_{t}+\sum_{a\in a_{t}}c_{t}\left(a\right)$

 Update exploration probability *p*_*t*+1_ using (7).

The following results show the plots of the reward functions for the model with an unsatisfied user, as described above. The plots show both the true reward *R*_*t*_/*t* as a function of the iteration number *t*, as well as the reward that the server believes to describe the user’s satisfaction, that is, ${\tilde{R}}_{t}/t$. We perform three simulations to illustrate the results above.

**Fig 3:**
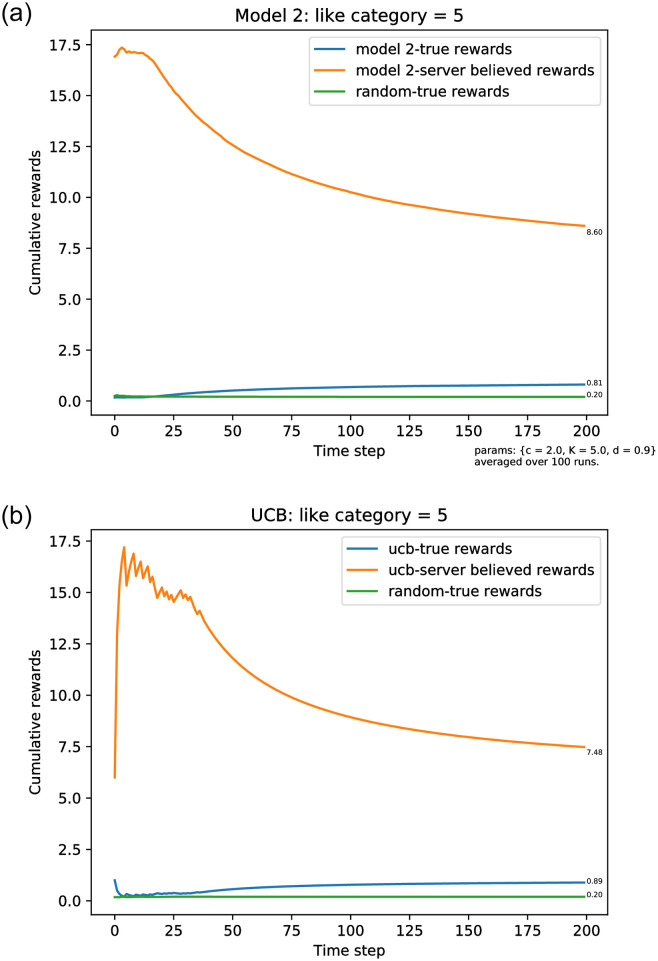
Simulation results for the true average reward rates where categories of items are presented in a fixed order and users continue clicking until seeing a liked item. The true average reward rate $({\hat{R}}_{t}/t),t=1,\ldots ,T$, where the server believes the average reward to be ${\tilde{R}}_{t}/t$. Here $A=100,\ell =20,a^{*}=5$. (a) Model 2 rewards, the exploration rate follows Eq (7). (b) The server uses the UCB policy.

Our first simulation assumes that the user only likes category a* = 5. As described above, there is a transition time *ν* before the server picks a* to be presented in the list. From that time onwards the server will continue to present this category, because the corresponding estimate *θ*_*t*_(a*) = 1 (we have assumed that the user always clicks on the preferred category when presented). However, all other categories presented in the list in front of a* will also have an estimate of *θ*_*t*_(*a*) = 1, *a* < a* provided that these categories have been shown at least once, because we assume that the user clicks on items in the order that they are presented. Because of occurrences of random exploration, eventually all five categories *a* ∈ {0, 1, …, 4} will appear in the list, so the believed reward by the server has a limit ${\text{lim}}_{t\to \infty }{\tilde{R}}_{t}/t=6$. But this is utterly incorrect. The user only likes category a* = 5, so the true reward has a limit *R*_*t*_/*t* → 1. This behaviour is clearly evident in Fig 3a.

Fig 3b shows the corresponding plots when using the UCB algorithm for this model. In our simulations, the user still clicks until they find a category they like. The only difference is that, instead of using the top-*ℓ* estimated *θ* values and random exploration, the server presents the list according to the top-*ℓ* estimated upper bounds. As the figure illustrates the asymptotic behavior is the same as with the *ϵ*_*n*_-greedy policy.

**Fig 4:**
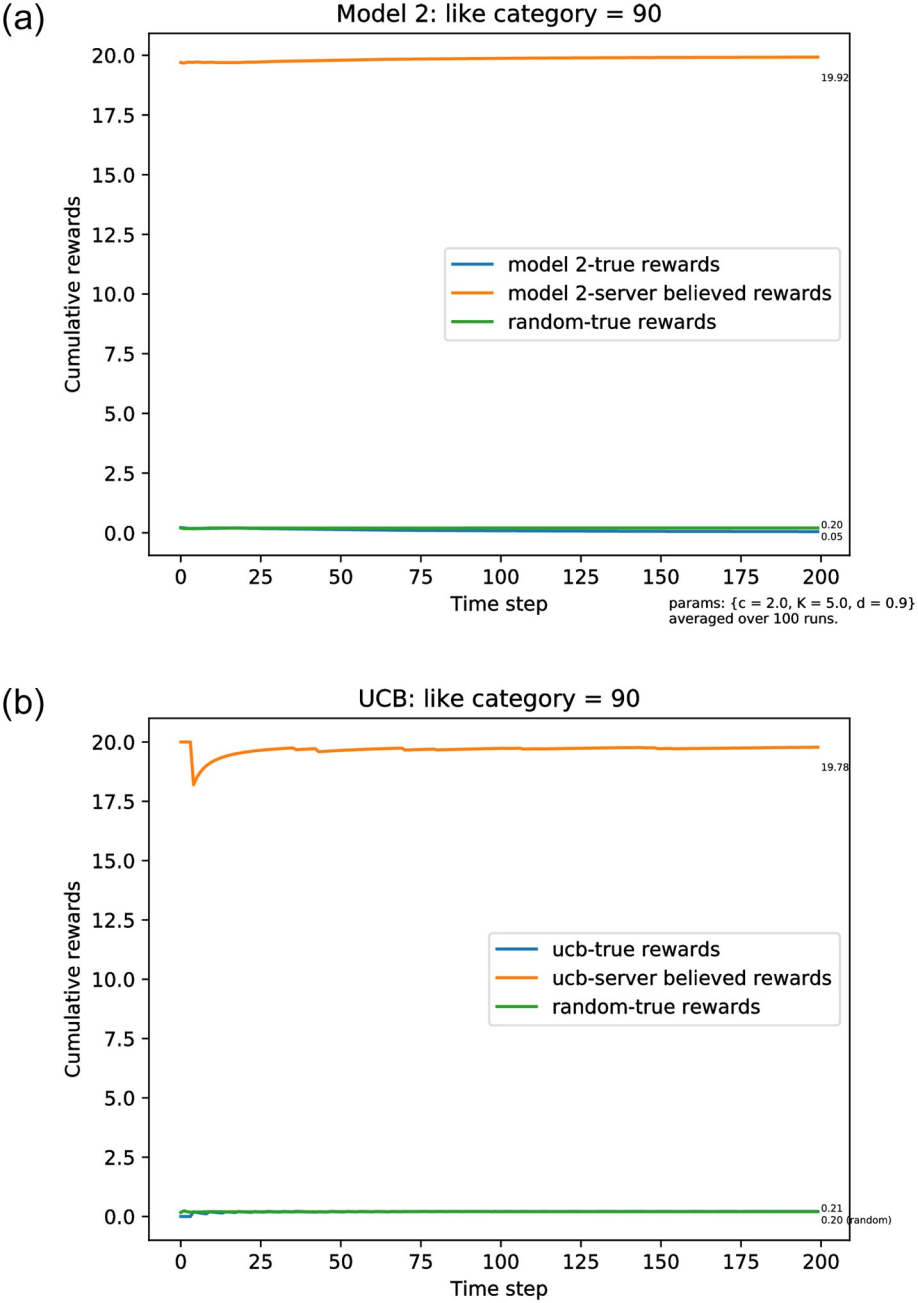
Simulation results for the true average reward rates where categories of items are presented in a fixed order and users continue clicking until seeing a liked item. The true average reward rate $({\hat{R}}_{t}/t),t=1,\ldots ,T$, where the server believes the average reward to be ${\tilde{R}}_{t}/t$. Here $A=100,\ell =20,a^{*}=90$. (a) Model 2 rewards, the exploration rate follows Eq (7). (b) The server uses the UCB policy.

To illustrate Proposition 3.2, we present in Fig 4a a second simulation when a* = 90 > *ℓ*. As predicted, the server eventually diverges from the true satisfaction, and, even if it has served the preferred category, it is fooled by the clicks on items served in front of the list. Eventually the server stops serving the preferred category during exploitation (and the exploration probability goes to zero). In the plot, it is evident that this happens very quickly. Because the list has *ℓ* = 20 items, the server believes that it is achieving the maximal satisfaction rate, that is, ${\text{lim}}_{t\to \infty }{\tilde{R}}_{t}/t=20$, while the user’s satisfaction rate is going to 0.

**Fig 5:**
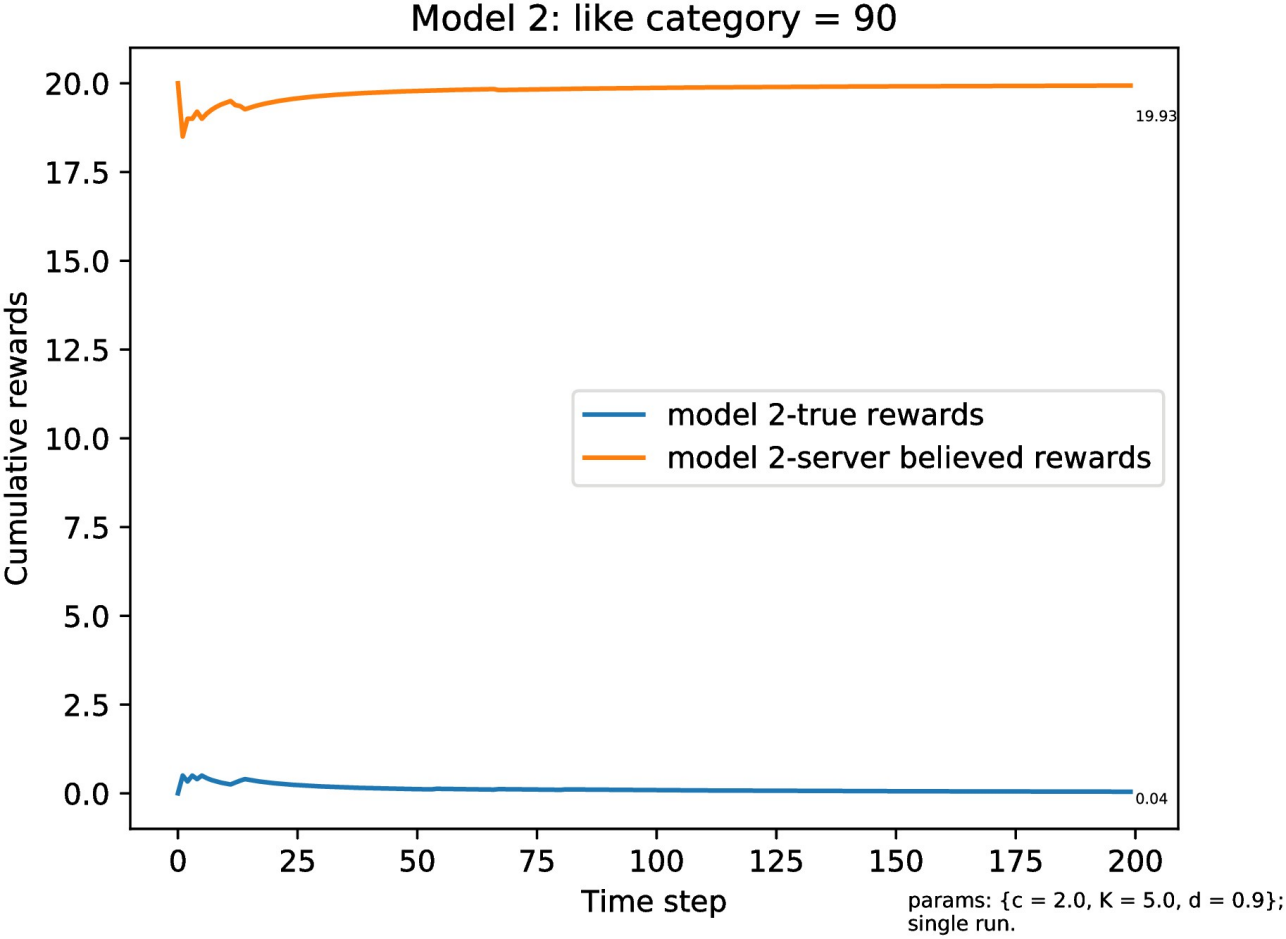
Simulation results of model 2 for a trajectory of true reward $({\hat{R}}_{t}/t),t=1,\ldots ,T$, where the server believes the reward to be ${\tilde{R}}_{t}/t$. Here A=100, *ℓ* = 20, and a* = 90.

Fig 4a shows the average rewards over 100 replications of the simulation when a* = 90, as discussed. Fig 5 shows a typical trajectory, demonstrating that, as expected, even when the correct category appears at an early time, it slowly gets replaced by items in other categories that are shown at the front of the list.

The plot shown in Fig 4b shows the corresponding rewards when the server uses the UCB policy. As expected, the asymptotic behavior is the same as with the *ϵ*_*n*_-greedy policy, illustrating again that the principles that we have established with out theoretical analysis are independent of the specific method for machine learning used by the server.

**Fig 6:**
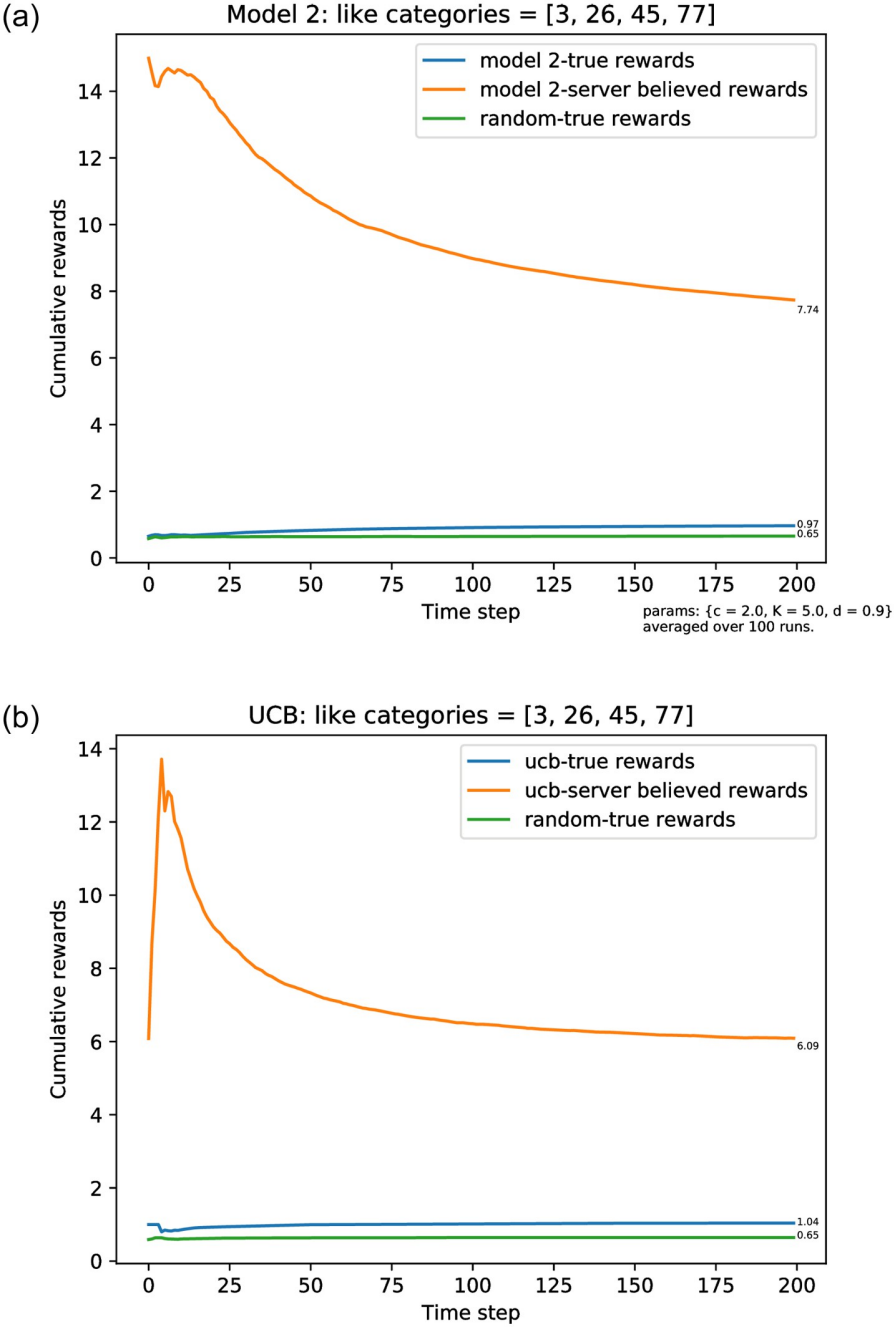
Simulation results for model 2 for the true average reward $({\hat{R}}_{t}/t),t=1,\ldots ,T$ and the server-believed average reward ${\tilde{R}}_{t}/t$. Here A=100,ℓ=20. The true satisfaction probabilities of all categories are zero, except for four categories: 3, 26, 45, and 77; their respective probabilities are *μ*(3) = 0.88, *μ*(45) = 0.54, *μ*(77) = 0.92, and *μ*(26) = 0.62. (a) Model 2 rewards, the exploration rate follows Eq (7). (b) The server uses the UCB policy.

Our third simulation is used to present a more realistic scenario, in constrast to the above, which is an extreme of only liking one category. It is illustrated in Fig 6a. In this simulation the user somewhat likes a few categories, and dislikes the rest. Specifically, here:

$$\begin{array}{c} \mu \left(a\right)=\{\begin{array}{cc} 0.88 & a=3\\ 0.62 & a=26\\ 0.54 & a=45\\ 0.92 & a=77\\ 0.00 & \text{all}\;\text{other}\;\text{categories} \end{array} \end{array}$$

Surprisingly, the plots in Fig 6a show behavior very similar to the extreme case. To understand the behaviour, in this case P(userlikesanitemincategory3)=0.88, meaning that the user will always click on this category when it is presented. With probability 0.88 the user will stop there; with probability 0.12 the user does not like the movie of category 3 that was served in the list, and proceeds to click on further items. As the other categories that the user likes are 26, 45, and 77, any category *a* < 26 served will be clicked, provided that either category 3 is not served or it is served but the movie was liked.

A simple analysis demonstrates that eventually, due to random exploration, all first 20 categories will be persistent. Because category 3 is the only one included, the user’s reward rate satisfies *R*_*t*_/*t* → 0.88, while the maximal rate would be obtained by including 3, 26, 45, and 77 in the served lists, giving 0.88 + 0.12(0.62 + 0.38(0.54 + 0.46 × 0.92)) = 0.9983. On the other hand, the server believes that the user likes all four of the first categories plus, with probability 0.12, all of the following 16 categories, yielding ${\tilde{R}}_{t}/t\to 4+0.12\left(16\right)=5.92$. The true optimal action would be to recommend items from categories (77, 3, 26, 45, …) in this order, where any other 16 categories can fill the lists.

However, there is no mechanism for the server to learn that the order of the items served is important for this user. This is the fundamental problem we are considering: that any user behaviour that lies outside the assumptions of the system can be undetectable, and lead to asymptotically catastrophic behaviour.

The final experiment in this section that we present is the result of applying the UCB policy for learning, for the same model as in Fig 6a. As before, we verify that the main asymptotic behavior is independent of the learning mechanism used to choose the recommendations.

Critically, these results show that although the server’s response is close to random, the server’s metrics show that it is doing well; in the presence of unanticipated behaviour an arbitrary degree of failure is invisible. In principle the response can even be worse than random, that is, the actual satisfaction rate (true reward) would be below that given by an arbitrary choice of item. This is evident in the (extreme) case where a* = 90, because a random recommender would persistently include this category with probability *ℓ*/*M* = 1/5, while the ‘smart’ algorithms eventually cease to present this category. This is also true for our mixed experiment for the same reason: the ‘smart’ recommender eventually only includes *a* = 3 in the served lists, but a random recommender would persistently also include categories 26, 45, and 77, thus increasing the average reward.

In our experiments, the user is not aware of the category that they like. Instead, they click until finding an item that is satisfactory. It may be argued that, in the case of Fig 3a, when the user only likes category a* = 5 they may eventually learn that the first five items presented are of no interest, and so would start clicking the sixth item presented. First, this is a situation where the user learns and changes their behavior, rather than the algorithm learning from the user to adapt its strategy. Second, and more importantly, we observe that were this the case the server would eventually update the estimated *θ*_*t*_(*a*), *a* < a* = 5 as being smaller than *θ*_*t*_(a*), so it will present items from category a* = 5 before position 6 in the list. Our user has already learnt to skip the first five items, so will again be in the position of clicking items that are disliked. There is no intrinsic way for the computer to learn the correct answer.

### 3.3 Hidden or silent categories

We now present a different scenario. Continuing with the analogy of the movie recommender system, we assume here that the server has already classified movies into *A* categories with labels A={0,…,A-1}, where ∥A∥=A.

We assume that the server behaves as follows. The parameter $\theta_{t}\left(\cdot \right)\in R^{+}$ represents the estimation of the user’s cumulative satisfaction by the server and a list ***m***_*t*_ = (*m*_*t*,1_, …, *m*_*t*,*ℓ*_) is served as follows. The number of items (movies) of each of the categories, denoted by ***L***_*t*_ = (*ℓ*_*t*,1_, …, *ℓ*_*t*,*A*_), is chosen according to a multinomial distribution MN (*ℓ*, ***p***_*t*_) where where

$$\begin{array}{c} p_{t}\left(a\right)=\frac{\theta_{t}\left(a\right)}{\sum_{a^{\prime }\in A}\theta_{t}\left(a^{\prime }\right)},\;a\in A. \end{array}$$ (10)

Recall that a multinomial random variable satisfies: *ℓ*_*t*,*a*_ = number of category *a* chosen for the list, with $\sum_{a=1}^{A}\ell_{t,a}=\ell .$ When a list is served, for each 1 ≤ *i* ≤ *ℓ* the actual item *m*_*t*,*i*_ served is a (random) movie from each of the chosen categories k∈A such that *ℓ*_*t*,*k*_ ≠ 0. For each item served, the category from the server’s list is unique; we use the notation *m*_*i*_ ∈ *a* to indicate that the *i*-th movie served belongs to category *a*. So, for example, if *ℓ*_*t*,1_ = 3 then three of the items in the served list belong to category 1, in which case the server presents three different movies from category 1.

The list presented is denoted as ***m***_*t*_ = (*m*_*t*,1_, …, *m*_*t*,*ℓ*_), chosen as follows. The first item *m*_*t*,1_ is of the first category that is present in the list ***L***_*t*_, that is,

$$\begin{array}{c} a_{t,1}=\text{min}\left(a:\ell_{t,a}>0\right),\;\;\text{and}\;m_{t,1}\in a_{t,1}. \end{array}$$

Then the list ***L***_*t*_ is updated by decreasing *ℓ*_*t*,*a*_ by one. Denote by $L_{t}^{\prime }$ the resulting modified list. The next item *m*_*t*,2_ is found using the same algorithm: the first category of the updated list that has a positive number, that is, $a_{t,2}=\text{min}\left(a:\ell_{t,a}^{\prime }>0\right),\;\;\text{and}\;m_{t,2}\in a_{t,2},$ and $\ell_{t,a}^{\prime }$ is decreased by one. The recursion is executed until the last movie is chosen. Note that, if the original number *ℓ*_*t*,*a*_ > 1, then consecutive movies presented in the list are of the same category.

A click on an item corresponds here to watching the movie, taken by the system to imply that the user liked the item served. Thus we use

$$\begin{array}{c} \theta_{t+1}\left(a\right)=\theta_{t}\left(a\right)+C_{t}\left(a\right), \end{array}$$ (11)

where *C*_*t*_(*a*) is the total number of clicks at time *t* of items in category *a* presented at *t* (see below for a model for the clicks). An item that has been clicked will not be chosen again by the server.

#### 3.3.1 Probability model for user’s behavior

The model corresponds to the situation where the user likes a particular category, but this *hidden category* is not one of the predefined categories identified by the server—and thus it is not discoverable feature for the machine learning algorithms. That is, the representation is incomplete.

We denote the hidden category as *α*. For example, the server may use a list such as ‘drama, comedy, family, thriller, horror, Oscar winner, documentary, sports’. However, the user likes only movies from Spain, Mexico, or Colombia. While α∉A, for every server category a∈A, it is likely that *a*∩*α* ≠ ∅, meaning that there are movies of the hidden category present in every one of the server’s defined categories.

Thus, when served a list of items ***m***_*t*_ = (*m*_*t*,1_, …, *m*_*t*,*ℓ*_), we have the *click model* that the user clicks on all of the items that belong to hidden category *α*. We denote *C*_*t*_(*a*) = ∑_(*i*:*m*_*t*,*i*_ ∈ *a*)_ *c*_*t*_(*m*_*t*,*i*_), where for *i* = 1, …, *ℓ*

$$\begin{array}{c} c_{t}\left(m_{t,i}\right)=\{\begin{array}{cc} 1 & \;\text{if}\;m_{t,i}\in \alpha \;\left(\text{user}\;\text{clicks}\;\text{on}\;\text{movie}\;m_{t,i}\;\text{at}\;\text{time}\;t\right)\\ 0 & \;\text{otherwise.}\; \end{array} \end{array}$$ (12)

We use the notation *f*_*t*_(*a*) for the fraction of *α* movies remaining in category *a* at time *t*, that is,

$$\begin{array}{c} f_{t}\left(a\right)=P\left(\text{User}\;\text{likes}\;\text{item}\;m\in m_{t}\;|\;m\in a\right) \end{array}$$ (13)

This probability will directly determine the model for the random variable *C*_*t*_(*a*). From our model, it follows that $P(C_{t}\left(a\right)=k\;|\;\ell_{t,a})$ is the probability that exactly *k* amongst the *ℓ*_*t*,*a*_ movies of category *a* served in the list are *α*-movies.

When *M* > >*m* a simple counting argument can be used to approximate the distribution of *C*_*t*_(*a*) by the following binomial distribution:

$$\begin{array}{c} P\left(C_{t}\left(a\right)=k\;|\;\ell_{t,a}\right)=\left(\begin{array}{c} l_{t,a}\\ k \end{array}\right)f_{t}{\left(a\right)}^{k}\;{\left(1-f_{t}\left(a\right)\right)}^{\ell_{t,a}-k},\;0\leq k\leq \ell_{t,a}. \end{array}$$ (14)

**Lemma 3.2.** *Under the assumption that the list is chosen according to the MN(ℓ, **p***_*t*_) *distribution (see* Eq (10)) and that (14) *holds, then*

$$\begin{array}{c} E\left[C_{t}\left(a\right)\;|\;F_{t}\right]=\ell (\frac{\theta_{t}\left(a\right)}{{\bar{\theta }}_{t}})\;f_{t}\left(a\right), \end{array}$$ (15)

*where* ${\bar{\theta }}_{t}=\sum_{a}\theta_{t}\left(a\right)$ *and* $F_{t}$ *is the sigma-field (or history) of the process {ℓ*_*n*,⋅_, *n* ≤ *t*}.

*Proof.* Fix the time *t*; we now drop this index from the notation. Because the served list with *ℓ* items chooses category *a* according to the multinomial distribution, then it follows that the number *ℓ*_*t*,*a*_ of movies from category *a* served at time *t* has a marginal distribution that is a binomial distribution, that is *ℓ*_*a*_∼ Bin (*ℓ*, ***p***_*t*_(*a*)). On the other hand, it follows from Eq (14) that $E\left[C_{t}\left(a\right)\;|\;\ell_{a}\right]=\ell_{t,a}f_{t}\left(a\right)$, which implies that, for any *a* = 0, …, *A* − 1, $E\left[C_{t}\left(a\right)\right]=E\left[E\left[C_{t}\left(a\right)\;|\;\ell_{t,a}\right]\right]=E\left[\ell_{t,a}f_{t}\left(a\right)\right]=\ell \;p_{t}\left(a\right)\;f_{t}\left(a\right).$ Use of Eq (10) now proves the assertion.

As we keep a constant amount *M* of items in each category and the user has already consumed *C*_*t*_(*a*) movies of category *a* at time *t*, our model assumes that the server outsources new *C*_*t*_(*a*) movies of the same category *a*. For simplicity, we also assume that the fraction of *α* movies added is the same as the original fraction *f*_0_(*a*). This leads to the update:

$$\begin{array}{c} f_{t+1}\left(a\right)=f_{t}\left(a\right)-\frac{C_{t}\left(a\right)}{M}+\frac{\xi_{t}\left(a\right)}{M}, \end{array}$$ (16)

where *ξ*_*t*_(*a*) has a binomial distribution Bin(*C*_*t*_(*a*), *f*_0_(*a*)) and it is independent of the other random variables. This follows because *ξ*_*t*_(*a*) represents the number of new *α*-movies in category *a* and we assume that the fraction of *α* movies in the (unbounded) pool of outside movies is the constant *f*_0_(*a*). Thus $E\left[\xi_{t}\left(a\right)\;|\;C_{t}\left(a\right)\right]=f_{0}\left(a\right)\;C_{t}\left(a\right)$. For simplicity, from here onwards we assume that *f*_0_(*a*) ≡ *f*_0_ is constant for all categories, and call $k=\frac{\left(1-f_{0}\right)}{M}$. When convenient, we use the approximate model for the updates of the fractions:

$$\begin{array}{c} f_{t+1}\left(a\right)=f_{t}\left(a\right)-k\;C_{t}\left(a\right) \end{array}$$ (17)

#### 3.3.2 Analysis and simulation results

We now proceed to describe how the server’s estimate *θ*_*t*_ and the user’s real preference *f*_*t*_ behave. It develops that the model is related to a modified predator–prey model, and will be a basis of our claim that the ‘learning’ algorithm actually works worse than a random recommender. The server updates its estimates using Eq (11) and the fractions decrease according to Eq (16), that is: *θ*_*t*+1_(*a*) = *θ*_*t*_(*a*) + *C*_*t*_(*a*) and $f_{t+1}\left(a\right)=f_{t}\left(a\right)-\frac{C_{t}\left(a\right)}{M}+\frac{\xi_{t}\left(a\right)}{M}$, where *ξ*_*t*_(*a*)∼ Bin(*C*_*t*_(*a*), *f*_0_).

The (vector-valued) stochastic process is the joint process {(*θ*_*t*_, *f*_*t*_);*t* = 1, 2, …}. To analyze the behavior, we consider first the conditional drifts, or ‘tendencies’ for the processes. (A process *x*_*t*_ has conditional drift $E[x_{t+1}-x_{t}\;|\;x_{t}]$, a quantity that is useful for assessing how the dynamics evolve.) Using the results of Lemma 3.2 yields

$$\begin{array}{cc} E[\theta_{t+1}\left(a\right)\;|\;F_{t}] & =\theta_{t}\left(a\right)+\ell (\frac{\theta_{t}\left(a\right)}{{\bar{\theta }}_{t}})\;f_{t}\left(a\right)\\ E[f_{t+1}\left(a\right)\;|\;F_{t}] & =f_{t}\left(a\right)-k\;\ell (\frac{\theta_{t}\left(a\right)}{{\bar{\theta }}_{t}})\;f_{t}\left(a\right). \end{array}$$

These finite difference equations already point at the key fact: the higher the value of *θ*_*t*_(*a*) the higher the chance of showing items in category *a*, which exhausts the remaining *α*-movies, thus decreasing *f*_*t*_(*a*) and the user’s satisfaction. However, the server increases *θ*_*t*_(*a*) instead of decreasing it. The system thus has similarities to a predator–prey model: the predator is the server (via *θ*_*t*_(⋅)) but, in consuming the resources (the *α*-movies), it depletes the prey (via *f*_*t*_(⋅)).

Remark We note that the update Eq (17) for *f*_*t*_ is not quite correct because in principle this could lead to negative values, so a truncation must be added to ensure that *f*_*t*_ ≥ 0 w.p.1. We study the general behavior, but truncate the updates; that is, our code uses *f*_*t*+1_(*a*) = max(0, *f*_*t*_(*a*) − *k C*_*t*_(*a*)).

In addition to describing the dynamics, we use the cumulative reward function

$$\begin{array}{c} R_{T}=E[\sum_{t=1}^{T}\sum_{a\in A}C_{t}\left(a\right)\;|\;F_{T}]=R_{T-1}+\sum_{a\in A}\ell (\frac{\theta_{t}\left(a\right)}{{\bar{\theta }}_{t}})\;f_{t}\left(a\right). \end{array}$$

This expression is a direct consequence of Lemma 3.2. It uses the fact that the expected instantaneous reward from category *a* (the expected number of clicks) is the probability that *a* is chosen $\ell (\theta_{t}\left(a\right)/\bar{\theta }\left(a\right))$ times the probability that the chosen movie is of category *α*.

Remark Unlike the model for the dissatisfied user, in this model the user clicks when they are happy with the item. In that sense, the satisfaction is indeed the expected number of clicks, so the reward above is correct. The difference between this model and Eq (3) is that in this case, because of the hidden preference, the probability of liking a category changes with time. In other words, *μ*(*a*) is not constant but instead is equal to *f*_*t*_(*a*).

Algorithm 3 is used for the simulation of hidden categories, as follows. Following the description above, given *θ*_*t*_ the server chooses the categories according to a multinomial random variable MN(*ℓ*, ***p***), using Eq (10) and this defines the list of movies presented at step *t*. The user then clicks on the movies that are of the hidden or silent category *α*. We simulate this using Lemma 3.2 so that *C*_*t*_(*a*)∼ Bin(*ℓ*_*t*,*a*_, *f*_*t*_(*a*)), which provides the immediate expected reward for each *a*: $E\left[C_{t}\left(a\right)\right]=\ell \;p_{t}\left(a\right)\;f_{t}\left(a\right)$. (This *conditional Monte Carlo approach* enables our simulation to be efficient, because it reduces variance.) The server updates *θ* with (11) and the fraction of *α*-movies is updated using Eq (17).

**Algorithm 3** Simulation algorithm for Hidden category

Initialize (read) A,ℓ, *f*_0_(*a*) = *f*_0_, *θ*_0_(*a*).

$R_{0}=0,p_{0}\left(a\right)=\theta_{0}\left(a\right)/\sum_{a^{\prime }}\theta_{0}\left(a^{\prime }\right)$ (from (10))

**for** t ≔ 1 to T **do**

 Served list: ***L***_*t*_∼ MN(*ℓ*, ***p***).

 User Feedback:

 **for** a∈A **do**

  *C*_*t*_(*a*)∼ Bin(*ℓ*_*t*,*a*_, *f*_*t*_(*a*)).

 Calculate expected reward: ${\hat{R}}_{t+1}={\hat{R}}_{t}+\sum_{a\in A}\ell \;p_{t}\left(a\right)\;f_{t}\left(a\right)$

 Server update: *θ*_*t*+1_(*a*) using (11)

 Fractions update: *f*_*t*+1_(*a*) using (17)

 Update probability ***p***_*t*+1_ using (10).

Our benchmark is the *random recommender*, for which the cumulative reward is simply

$$\begin{array}{c} R_{T}^{*}=\sum_{t=0}^{T-1}\frac{\ell }{A}\sum_{a\in A}f_{t}\left(a\right), \end{array}$$

because each category is chosen at random (with replacement) from the *A* possibilities to be served in the list of *ℓ* items. That is, this system behaves using constant *θ*_*t*_(*a*).

We can further illustrate the benchmark behavior of the random policy. Here, because categories *a* are chosen always at random from the set A to fill the list of *ℓ* movies, we have $E\left[C_{t}\left(a\right)\;|\;F_{t}\right]=\left(\ell /A\right)f_{t}\left(a\right).$ Using *f*_*t*+1_(*a*) = *f*_*t*_(*a*) − *k C*_*t*_(*a*), for any a∈A we have

$$\begin{array}{c} E\left[f_{t+1}\left(a\right)\;|\;F_{t}\right]=f_{t}\left(a\right)-(\frac{k\;\ell }{A}\;f_{t}\left(a\right))=f_{t}\left(a\right)(1-\frac{k\;\ell }{A}). \end{array}$$

Therefore the average fraction of *α*-movies of the random recommender decreases geometrically fast, we have $E\left[f_{t}\left(a\right)\right]=f_{0}{\left(1-k\ell /A\right)}^{t}$, and it is the same for all *a* (because *f*_0_(*a*)≡*f*_0_ is a constant). This yields

$$\begin{array}{c} R_{T}^{*}=\sum_{t=0}^{T-1}\ell \;E\left[f_{t}\left(a\right)\right]=\ell \;f_{0}\sum_{t=1}^{T}{\left(1-\frac{k\ell }{A}\right)}^{t}=\frac{1-{\left(1-k\ell /A\right)}^{T}}{\left(k\ell /A\right)}. \end{array}$$ (18)

We note that when simulating the random recommender, the only difference from Algorithm 3 is that there is no update on *θ*_*t*_, so that ***p***_*t*_ is constant.

**Fig 7:**
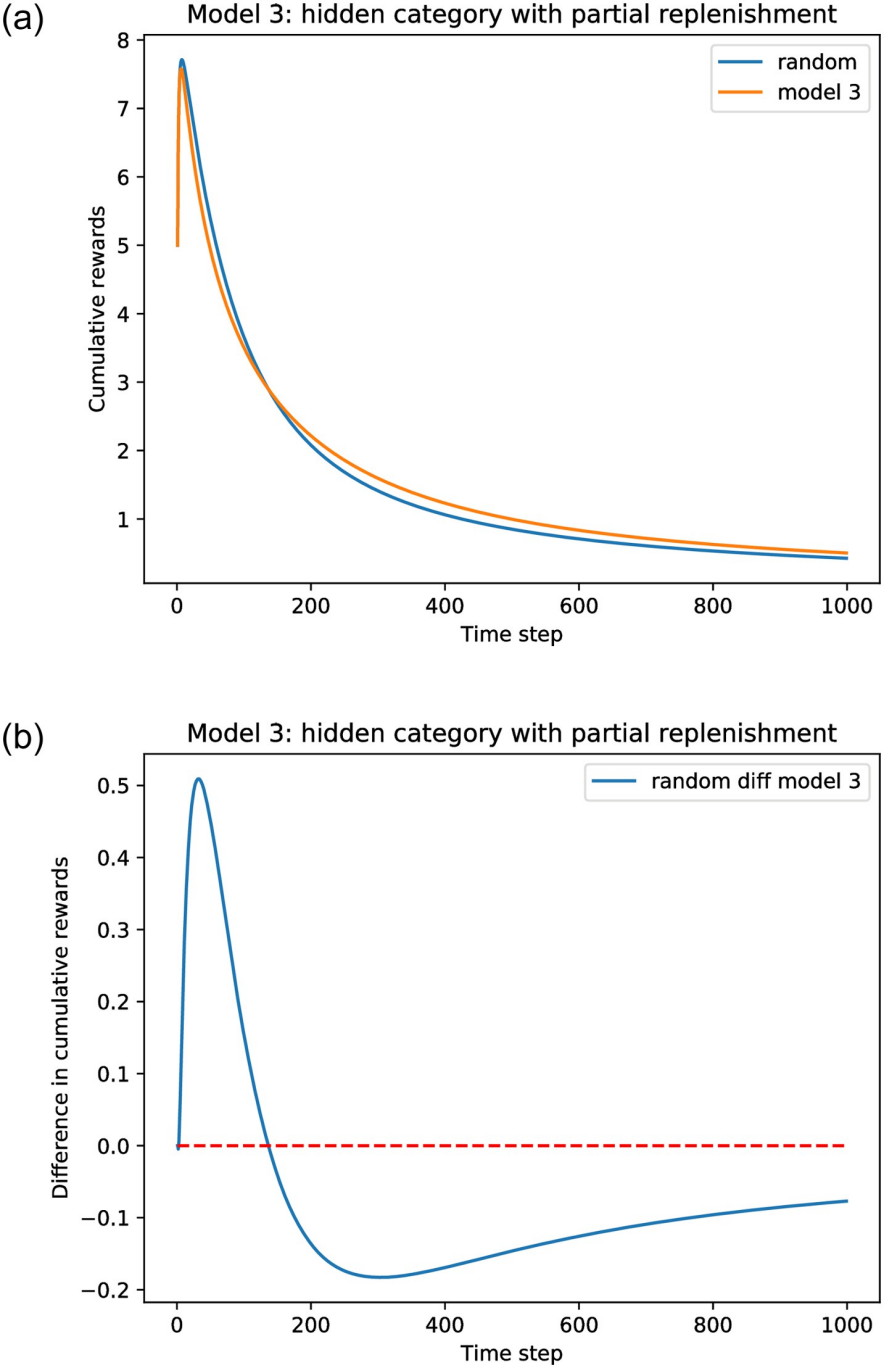
Simulation results comparing Model 3 with a random recommender. *A* = 100, list size *l* = 20, and *k* = 0.01. The original fraction *f*_0_(*a*) = 0.5 and *θ*_0_(*a*) = 0.5 for all categories.

Fig 7 shows the the cumulative rewards of the model with hidden categories. For our simulations we initialize all fractions *f*_0_(*a*) = *f*_0_ = 0.5. The smart recommender (explained below) follows the multinomial policy for the lists, and updates according to Eqs (11) and (17), as indicated in the pseudo-code for Algorithm 3.

**Fig 8:**
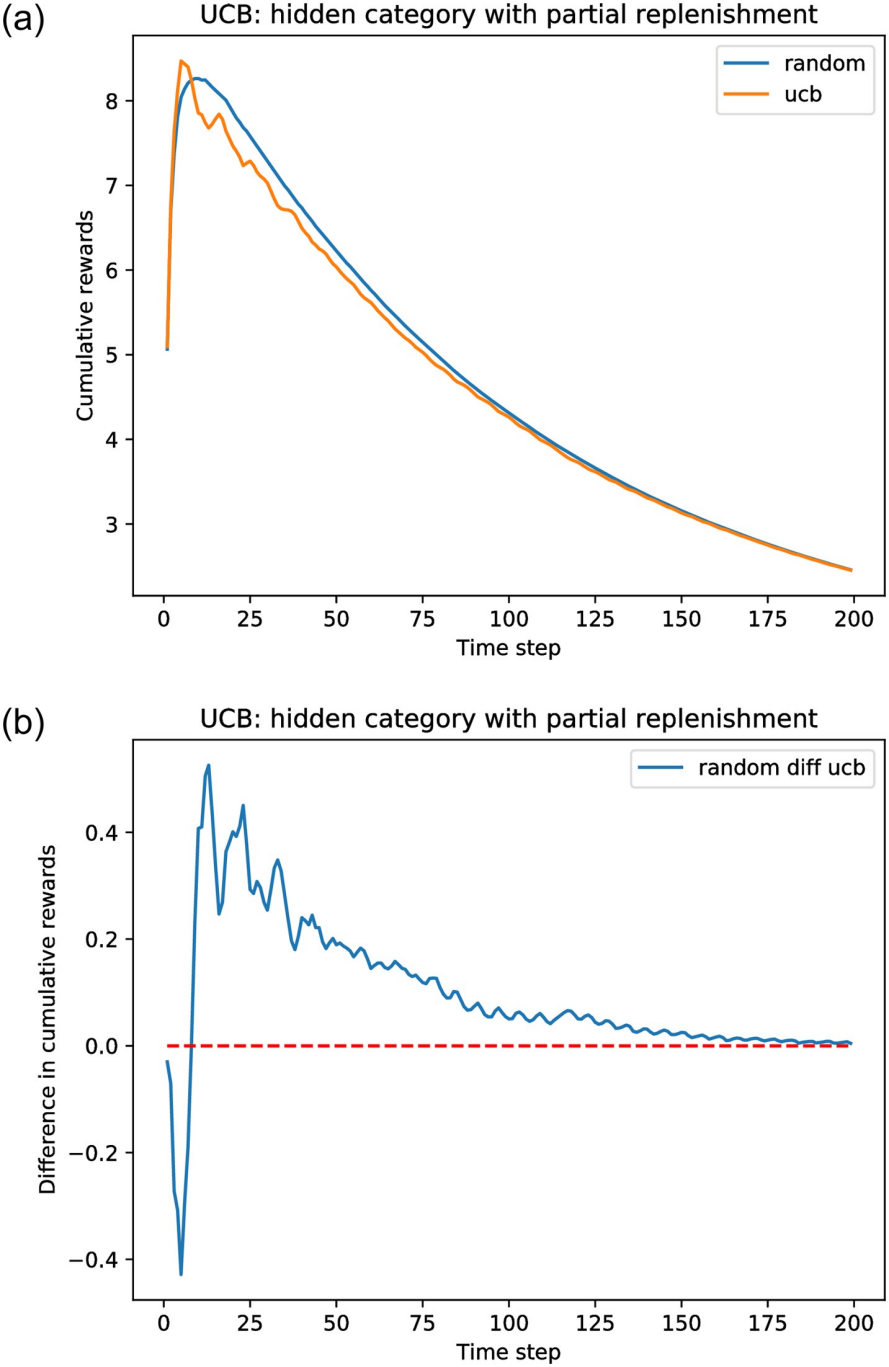
Simulation results comparing Model 3 under a UCB policy with a random recommender. A random recommender ignores user clicks when recommending categories; that is, *θ*_0_(*a*) is never updated. *A* = 100, list size *l* = 20, and *k* = 0.01. The original fraction *f*_0_(*a*) = 0.5 and *θ*_0_(*a*) = 1.0 for all categories. (a) Cumulative rewards. (b) Cumulative reward difference.

We call our simulation model for the learning scheme the *smart* recommender. Here we initialize all *θ*_0_(*a*) = 0.5 and all *f*_0_(*a*) = 0.5 so that there is no initial bias toward any given category. We refer to this as the *uniform* case. Other parameters have the same values as before. Fig 7 shows that even in the case that the learning has no initial bias, it is still worse than the random recommender. The plots were obtained performing 100 replications of the simulations to calculate the average rewards. We observe that for this model there is partial replenishment of the items served, because only the movies that are liked are replaced. As a consequence, because the user exhausts only the category *α* items, there is a natural depletion of the resources, until the fraction of *α* movies left is close to zero and both uniform and random recommender yield a near-zero instantaneous reward. Similar outcomes occur under the UCB policy, as shown in Fig 8.

#### 3.3.3 Full replenishment

As a further alternative, we now assume that the feedback from the user is a ‘thumbs-up’ or ‘thumbs-down’ binary action. Thus, when presented with a list ***L***_*t*_ of *ℓ* items (movies) the user provides a binary rank for each item. The user still clicks on all of the liked items, so the model for the clicks *C*_*t*_(*a*) is the same as before.

However, it is now more natural to assume that the server replaces all *ℓ* items served: that is, it removes all *ℓ*_*t*,*a*_ movies for category *a* that were presented to this user and instead shows new movies of that category, regardless of whether the user liked them or not. The rationale is that the user will have already judged (and perhaps consumed) all items in the previous list.

Eqs (12) and (13) still hold, and Lemma 15 is still valid for this model. The only difference is in Eqs (16) and (17), because now the server will also replace not-*α* movies and it is likely that categories with small *f*_*t*_(*a*) increase their fraction of *α*-movies.

In this model we have:

$$\begin{array}{c} f_{t+1}\left(a\right)=f_{t}\left(a\right)-\frac{C_{t}\left(a\right)}{M}+\frac{\zeta_{t}\left(a\right)}{M}, \end{array}$$ (19)

where now *ζ*_*t*_(*a*)∼ Bin(*ℓ*_*t*,*a*_, *f*_0_(*a*)) is the number of new movies that belong to hidden category *α*. In addition to Lemma 3.2 we will use the fact that $E\left[\zeta_{t}\left(a\right)\;|\;F_{t}\right]=\ell \;p_{t}\left(a\right)\;f_{0}\left(a\right).$

We first address the case of the random recommender.

**Proposition 3.3.** *Assume that the recommended list is chosen at random (with replacement) from the A categories. Under* Eq (19), *for any category* a∈A *the stochastic process of the fractions* {*f*_*t*_(*a*), *t* = 0, 1, …} *of α-movies in category a converges in distribution as M* → ∞ *to the solution of the ordinary differential equation*

$$\begin{array}{c} \frac{d}{dt}x\left(t\right)=f_{0}\left(a\right)-x\left(t\right), \end{array}$$ (20)

which has a unique limit (as t → ∞) and stable point x* = *f*_0_(*a*).

*Proof*. The proof is in an Appendix.

Before we provide the intuitive interpretation of the result, we state without proof the following result (which follows from Theorem 6.1 of Vázquez-Abad and Heidergott [15]), which provides a Functional Central Limit Theorem for the target tracking process above.

**Proposition 3.4**. *Consider the model in Proposition 3.3. Fix the category a and call* $U^{\in }\left(t\right)=\sqrt{\in }\left(x^{\in }\left(t\right)-f_{0}\left(a\right)\right)$. *Then as ϵ* → 0, *U*^*ϵ*^(⋅) *converges in distribution to the Orstein-Uhlenbeck process*

$$\begin{array}{c} dU(t)=-U(t)\;dt+\sigma \;dW(t), \end{array}$$

*where W*(⋅) *is a standard Brownian motion and* $\sigma^{2}=V\text{ar}\left[\zeta_{n}\left(a\right)-C_{n}\left(a\right)\;|\;f_{n}\left(a\right)=f_{0}\left(a\right)\right)]$.

The interpretation of the two results above is that, for sufficiently small (but constant) *ϵ*, the process {*f*_*n*_(*a*)} hovers around the stable limit *f*_0_(*a*) and has approximately normally distributed errors around this limit. For our study, this means that the full replenishment policy targets a constant fraction of *α*-movies, so they do not deplete (with probability 1) as before.

We now turn to the discussion of the multinomial (smart) strategy under full replenishment. The process for this model is more complicated, following Eq (11) for the updates, Eq (10) to define the probability parameter for the multinomial, and Eq (19) for the update of fractions under full replenishment. It follows that

$$\begin{array}{c} E\left[\theta_{t+1}\left(a\right)\;|\;F_{t}\right]=\theta_{t}\left(a\right)+\ell \;p_{t}\left(a\right)\;f_{t}\left(a\right);\;E\left[f_{t+a}\left(a\right)\;|\;F_{t}\right]=f_{t}\left(a\right)+\frac{\ell }{M}\;p_{t}\left(a\right)\left(f_{0}-f_{t}\left(a\right)\right). \end{array}$$

Looking only at the evolution of the fractions for any given a∈A, it is still the case that the only stable points correspond either to ***p***_*t*_(*a*) = 0, yielding *f*_*t*_(*a*) = 0 or *f*(*a*) = *f*_0_. If the estimates are all initialized with non-zero values then *θ*_*t*_(*a*)>0 always, excluding the first scenario. When ***p***_*t*_(*a*)>0 the dynamics of the fractions correspond to a target tracking algorithm with slowed down dynamics by the factor ***p***_*t*_(*a*). The behavior of the process of the fraction will be similar to the random case, albeit with different amplitude in oscillations around *f*_0_. However it is notable here that the tendency of the smart algorithm is still to increase the likelihood of appearance of category *a*, while for this same category the likelihood of containing *α*-movies has decreased. Further, if a category *a* has few *α*-movies left at time *t*, it is likely that *C*_*t*_(*a*) is null (or smaller than for other categories served); but all of the movies served from that category get replenished by new movies, thus increasing the fraction *f*_*t*+1_(*a*), while the server has decreased its estimate for *θ*_*t*_(*a*) thus decreasing the probability of serving this category at *t* + 1. This explains why the smart strategy is expected to perform even less well than the random recommender.

**Fig 9:**
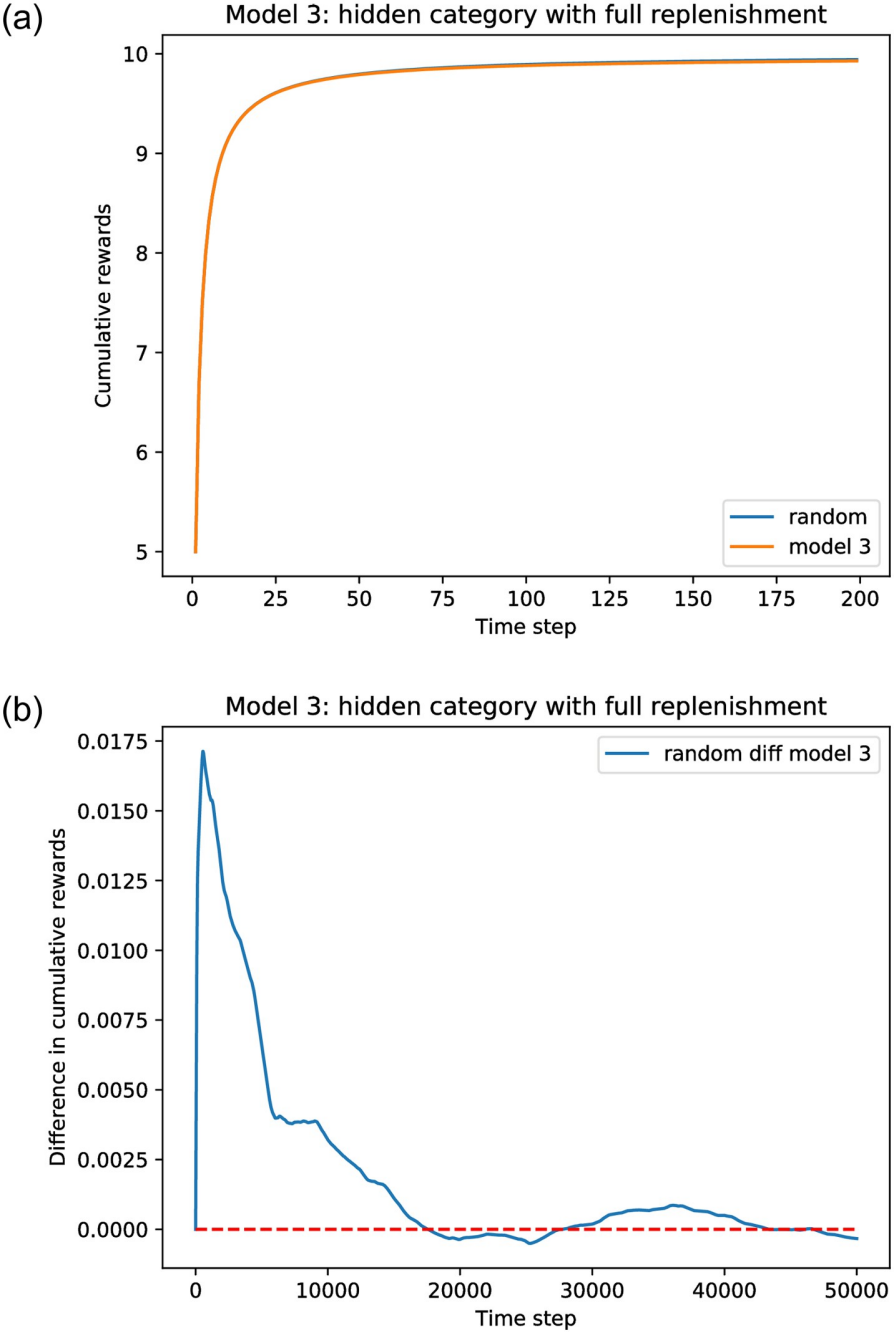
Simulation results for full replenishment, comparing Model 3 with a random recommender. *A* = 100, list size *l* = 20. The original fraction *f*_0_(*a*) = 0.5 and *θ*_0_(*a*) = 0.5 for all categories. (a) Cumulative rewards. (b) Cumulative reward difference.

Fig 9 shows simulation results for the smart recommender with full replenishment. The tendency to return to the mean for the random recommender can be clearly seen. The smart recommenders still perform less well than the random. Although it is occasionally superior, this superiority is not persistent. Incompleteness of representation has, again, led to failures of recommendation that the system cannot observe.

**Fig 10:**
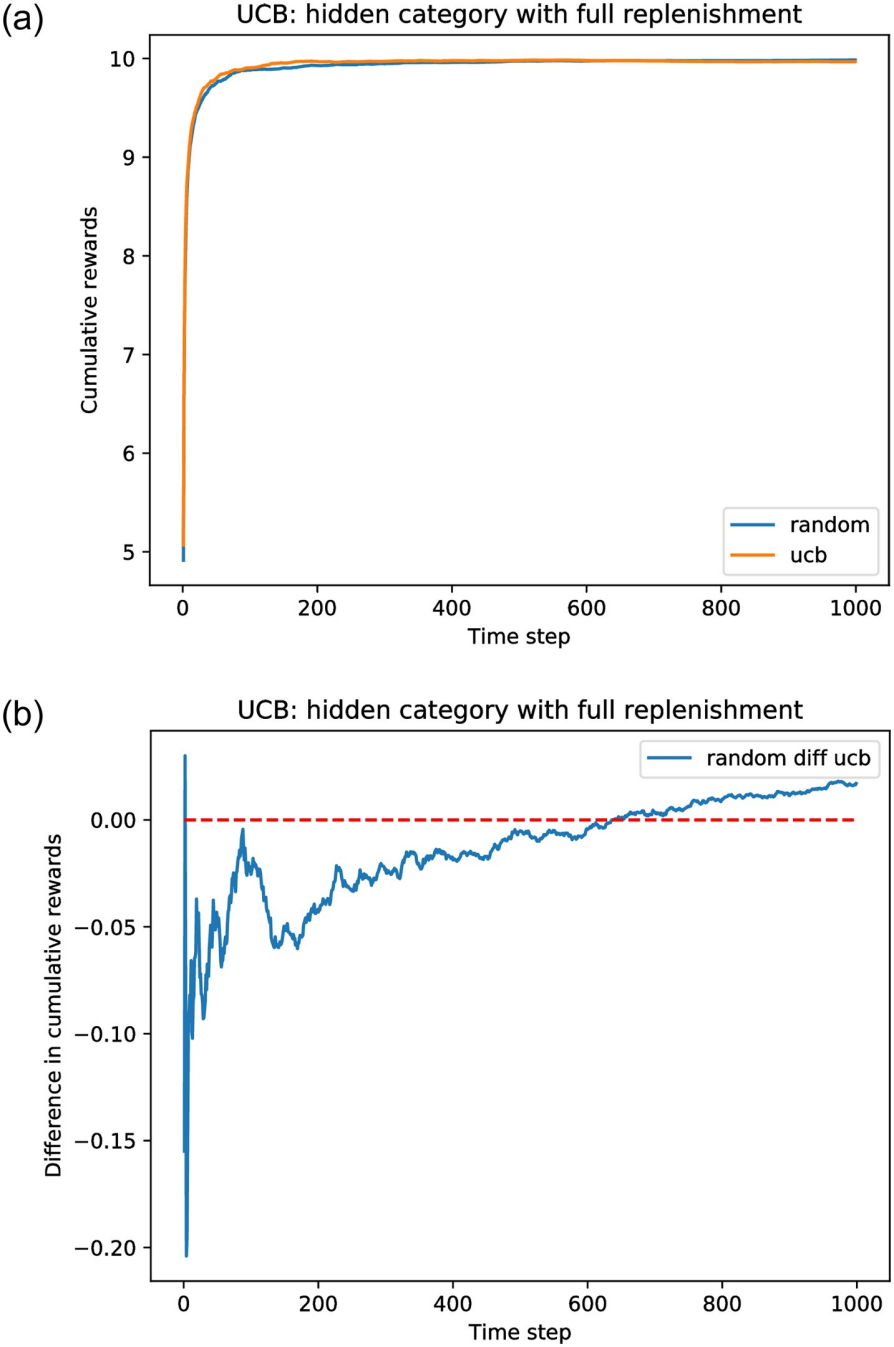
Simulation results for full replenishment, comparing Model 3 under UCB policy with a random recommender. *A* = 100, list size *l* = 20. The original fraction *f*_0_(*a*) = 0.5 and *θ*_0_(*a*) = 0.5 for all categories. (a) Cumulative rewards. (b) Cumulative reward difference.

As a final illustration, Fig 10 shows simulation results for the smart recommender under a UCB policy with full replenishment. The results are comparable to a random recommender.

## 4 Discussion and conclusions

We have explored the implications for proxy-driven machine learning of the situation where the real world exhibits behaviours that were not anticipated by the system designers. Using the relatively straightforward case of a single-user personalised recommendation system, we show that if the representation of the data—describing either the items being recommended or the user behaviour—is incomplete, then the resulting performance can be not just sub-optimal but no better than random.

In our case studies, we explore representational incompleteness in two ways, considering a user with unanticipated behaviour and the presence of unannotated categories. Under certain assumptions behaviour can even be worse than random, when for example the user’s preferences and the system’s inaccurate attempts to satisfy them are based on inconsistent premises that come into tension with each other.

The possibility of random or even worse-than-random behaviour may be unsurprising to some readers. However, it highlights two underlying issues that are widely neglected in exploration and deployment of machine learning methods. One is that the poor behaviour is invisible to the system; the metrics may well report that it is performing well. The other is that the effectiveness in practice of a machine learning method is limited by the completeness of the data representation. It is well known that erroneous data, or missing items, can lead to error; there is however no literature of which we are aware that explores the fact that the representation itself is a limitation.

Errors in data can in principle be corrected, and missing items replaced; but representations are innately incomplete and cannot be as rich as the entities they describe. Even the concept of category, which is crucial to the ability to gather ‘like’ items together, can be a poor fit to the natural world. That is, any learning algorithm applied to a rich human context has incomplete knowledge: it can only observe a pre-defined set of behaviours, make use only of specified features, and optimise only against known metrics. The algorithm cannot make use of or adapt to information of which it is unaware. As has been observed in other contexts, most notably with regard to Goodhart’s law (see for example Manheim and Garrabrant [16]), the resulting performance can be absurdly divergent from what was intended. Our results illustrate that there is a risk that a learning system will fail, not due to poor design, but because there are inherent limitations to automated systems that engage with human processes, and the system will be unaware of the extent of the failure.

Wherever there is a representation gap, there is a potential for unobserved failure of unbounded magnitude. Understanding of when such failures are arising, and of how wide the gaps are between representation and reality, is critical to reliable deployment of machine learning systems.

## 5 Appendix: Proof of Proposition 3.1

First, given that the server chooses to explore at time *t*, an action that happens with probability *p*_*t*_ > 0, using basic combinatorial arguments,

$$\begin{array}{c} P\left(a^{*}\notin a_{t}\;|\;\text{exploration}\right)=\frac{\left(\begin{array}{c} A-1\\ l \end{array}\right)}{\left(\begin{array}{c} A\\ l \end{array}\right)}=\frac{A-\ell }{A}\Rightarrow P\left(a^{*}\in a_{t}\;|\;\text{exploration}\right)=\frac{\ell }{A}. \end{array}$$

Let $X_{k}=1_{\left\{a^{*}\in a_{k}\right\}}$. Then for all *k* such that *k* < *ν*, $P\left(X_{k}=1\right)=P\left(a^{*}\in a_{k}\;|\;\text{exploration}\right)\;p_{k}$. That is, it can be seen that $P\left(X_{k}=1\right)=p_{k}\;\frac{\ell }{A}$, because with probability (1 − *p*_*k*_) the system will recommend the top *ℓ* categories of *θ*_*k*_ and a* is not there (we are considering *k* < *ν*, so *θ*_*k*_(a*) = 0). Because the variables {*X*_*k*_;*k* = 1, 2, …} are independent, we have

$$\begin{array}{c} P\left(\nu >k\right)=\prod_{j=1}^{k}P\left(X_{j}=0\right)=\prod_{j=1}^{k}(1-p_{j}\frac{\ell }{A}). \end{array}$$

Finally, using the identity $E\left[\nu \right]=\sum_{k=1}^{\infty }P\left(\nu \geq k\right)$ we obtain the result (9).

## 6 Appendix: Proof of Proposition 3.2

Every time that the iteration corresponds to an exploration, items are chosen at random. As discussed, once a* is chosen the estimate will be correct: *θ*_*t*_(a*) = 1 for all *t* > *ν*. Given the server algorithm, if an item *a*′ < a* is served at time *t*, then *θ*_*k*_(*a*′) = 1 for all *k* > *t*. This happens because the item is always presented before a* and the click model assumes that all items are clicked until reaching a* or the list is exhausted. Therefore, the server’s update in (5) as the sample mean number of clicks for item *a*′ will always remain at one unit. Because *p*_*t*_ > 0, eventually *ℓ* items with smaller labels than a* will be chosen during exploration. Call this time *τ*. It is a dual of the ‘surfacing’ time because it is the time when a* will no longer ever be served on exploitation iterations: all other smaller labels bump out the item a*. That is, call *τ* = min(*t*:max_*i*_(***a***_*t*,*i*_)<a*), then by the server’s policy and under the click model above, the top *ℓ* items according to *θ*_*k*_, *k* > *τ* will not contain a*. Because of random drawings, the stopping time *τ* is finite w.p.1.

## 7 Appendix: Proof of Proposition 3.3

For this proof we will use the notation *n* for discrete (iteration) times, and *t* for continuous time. Fix any category *a*, call *ϵ* = *ℓ*/*AM*, and re-write (19) as

$$\begin{array}{c} f_{n+1}\left(a\right)=f_{n}\left(a\right)+\epsilon (\frac{\zeta_{n}\left(a\right)-C_{n}\left(a\right)}{\ell /A}), \end{array}$$

which is a stochastic approximation algorithm known as ‘target tracking’, also called a ‘return to the mean’ recursion. To see this, note that for the random recommender, the conditional drift is $E\left[f_{n+1}\left(a\right)-f_{n}\left(a\right)\;|\;F_{n}\right]=\in \;\left(f_{0}\left(a\right)-f_{n}\left(a\right)\right).$ Intuitively, if *f*_*n*_(*a*)>*f*_0_(*a*) then the expected change is negative, while if *f*_*n*_(*a*)<*f*_0_(*a*), then it is positive. Define *x*^*ϵ*^(*t*) as the piecewise constant interpolation (stochastic) process *x*^*ϵ*^(*t*) = *f*_*n*_(*a*), for all *t* ∈ [*nϵ*, (*n* + 1)*ϵ*). Let *m*(*t*) = ⎣*t*/*ϵ*⎦ denote the number of iterations up to time *t*. Then *x*^*ϵ*^(*t*) = *f*_*m*(*t*)_(*a*). The *feedback* *Y*_*n*_ ≡ *A*(*ζ*_*n*_(*a*) − *C*_*n*_(*a*))/*ℓ* is a bounded random variable, because both *ζ*_*n*_(*a*), *C*_*n*_(*a*) ≤ *ℓ* w.p.1. By our previous calculations, calling $F_{n}$ the history of the process up to iteration *n*, $E\left[Y_{n}\;|\;F_{n}\right]=f_{0}\left(a\right)-f_{n}\left(a\right).$ The problem satisfies all the conditions in Theorem 4.3 of the Martingale noise model in Heidergott and Vázquez-Abad [15], which establishes the result. This reference is at present under revision by the publisher.

## 8 Appendix: Proof of Proposition 3.4

This appendix provides the proof of Proposition 3.4, following the techniques in Vázquez-Abad and Heidergott [15]. Uniform boundedness of {*Y*_*n*_} is a sufficient condition to ensure compactness of the stochastic processes {*x*^*ϵ*^(⋅);*ϵ* > 0} in the probability sense, called ‘tightness’. Thus every *ϵ*-sequence has a further weakly convergent (using the standard terminology, weak convergence is the same as convergence in distribution) subsequence and all limit points (in the sup-norm) are Lipschitz continuous processes w.p.1.

Consider one such weakly convergent subsequence with *ϵ* → 0 and call $\bar{x}\left(\cdot \right)$ the corresponding limit, which is Lipschitz continuous w.p.1. By construction, $x^{\in_{k}}\left(t+s\right)=x^{\in }\left(t\right)+\sum_{n=m(t)}^{m(t+s)}\in \;Y_{n}.$ Call ${\tilde{F}}_{t}$ the *σ*-algebra (or history) of the continuous time process *x*^*ϵ*^(⋅). It follows that ${\tilde{F}}_{t}\equiv F_{m(t)}$. Conditioning on ${\tilde{F}}_{t}$ we obtain

$$\begin{array}{c} E[x^{\epsilon }\left(t+s\right)-x^{\epsilon }\left(t\right)\;|\;{\tilde{F}}_{t}]\\ =E[\sum_{n=m(t)}^{m(t+s)}\epsilon \;\left(f_{0}\left(a\right)-f_{n}\left(a\right)\right)\;|\;F_{m(t)}]\\ \approx E[\int_{t}^{t+s}\left(f_{0}\left(a\right)-x^{\epsilon }\left(u\right)\right)\;du,\;|\;{\tilde{F}}_{t}] \end{array}$$

where we have used the integral representation of the sum, and the approximation error is uniformly bounded and is of order O(∈). Define the process: $M^{\in }\left(t\right)\equiv x^{\in }\left(t\right)-x^{\in_{k}}\left(0\right)-\int_{0}^{t}\left(f_{0}\left(a\right)-x^{\in }\left(u\right)\right)\;du,$ then it follows that along the weakly convergent subsequence, the corresponding limit process *M*(*t*) = lim_*k* → ∞_ *M*^*ϵ*^(*t*) is a w.p.1-Lipschitz continuous martingale, with initial value *M*(0) = 0, which implies that *M*(*t*) is a constant w.p.1. This, along the limit of such convergent subsequence satisfies

$$\begin{array}{c} \bar{x}\left(t+s\right)-\bar{x}\left(t\right)=\int_{t}^{t+s}\left(f_{0}\left(a\right)-\bar{x}\left(u\right)\right)\;du. \end{array}$$

which is equivalent to the solution of the ODE in Eq (20). This completes the proof, observing that this ODE has a unique solution for each initial condition, so this limit is independent of the chosen convergent subsequence. Using the standard compactness argument, this implies that the original sequence of stochastic processes converges in distribution to the solution of Eq (20).
